# A novel computational strategy for DNA methylation imputation using mixture regression model (MRM)

**DOI:** 10.1186/s12859-020-03865-z

**Published:** 2020-12-01

**Authors:** Fangtang Yu, Chao Xu, Hong-Wen Deng, Hui Shen

**Affiliations:** Center for Bioinformatics and Genomics, Department of Biostatistics and Data Science, School of Public Health and Tropical Medicine, Tulane University, New Orleans, LA 70112 USA

**Keywords:** Methylation, Imputation, Mixture of regression models, Epigenomic association studies

## Abstract

**Background:**

DNA methylation is an important heritable epigenetic mark that plays a crucial role in transcriptional regulation and the pathogenesis of various human disorders. The commonly used DNA methylation measurement approaches, e.g., Illumina Infinium HumanMethylation-27 and -450 BeadChip arrays (27 K and 450 K arrays) and reduced representation bisulfite sequencing (RRBS), only cover a small proportion of the total CpG sites in the human genome, which considerably limited the scope of the DNA methylation analysis in those studies.

**Results:**

We proposed a new computational strategy to impute the methylation value at the unmeasured CpG sites using the mixture of regression model (MRM) of radial basis functions, integrating information of neighboring CpGs and the similarities in local methylation patterns across subjects and across multiple genomic regions. Our method achieved a better imputation accuracy over a set of competing methods on both simulated and empirical data, particularly when the missing rate is high. By applying MRM to an RRBS dataset from subjects with low versus high bone mineral density (BMD), we recovered methylation values of ~ 300 K CpGs in the promoter regions of chromosome 17 and identified some novel differentially methylated CpGs that are significantly associated with BMD.

**Conclusions:**

Our method is well applicable to the numerous methylation studies. By expanding the coverage of the methylation dataset to unmeasured sites, it can significantly enhance the discovery of novel differential methylation signals and thus reveal the mechanisms underlying various human disorders/traits.

## Background

DNA methylation is one of the most important epigenetic marks in the human genome, during which a methyl group (–CH_3_) is added to the C-5 position of a cytosine of DNA. In mammals, more than 98% of DNA methylation occurs in the context of neighboring cytosine and guanine nucleotides (CpGs) in somatic cells, while as much as a quarter of all methylation appears in a non-CpG context in embryonic stem cells (ESCs) [1, 2]. DNA methylation plays a crucial role in transcriptional regulation. Typically, the occurrence of methylation in the promoter region of a gene will suppress the transcription of the gene, while the occurrence of methylation in the gene bodies is commonly associated with transcriptional activation [3, 4]. The significance of DNA methylation mediated epigenetic regulation has been well established by biological functional studies on embryonic development, genomic imprinting, X-chromosome inactivation, and the pathogenesis of various human disorders [5].

Recent advances in high-throughput microarray and sequencing techniques have made it possible to measure DNA methylation level on a genome-wide scale in a large number of samples thus facilitate association studies of DNA methylation with the phenotype of interest, i.e., EWAS (epigenome-wide association study) [6]. By performing EWAS, researchers have identified differentially methylated CpGs (DMCs), regions (DMRs), or genes associated with various diseases, including cancer [7], Alzheimer’s disease [8], rheumatoid arthritis [9], and diabetes [10]. The gold standard for DNA methylation measurement is whole-genome bisulfite sequencing (WGBS), which can comprehensively quantify ~ 26 million of the 28 million CpGs in the human genome [11]. However, it is still cost-prohibitive to apply WGBS to large-scale studies [12]. On the other hand, the commonly used cost-effective DNA methylation measurement methods only cover a small portion of the human genome. For example, the Illumina Infinium HumanMethylation-27 and -450 BeadChip arrays (27 K and 450 K arrays) and reduced representation bisulfite sequencing (RRBS) method only measure approximately 0.1, 2, and 8–10% of the total CpG sites, respectively [11]. The low genome coverage of methylation data from these techniques considerably limited the scope of the DNA methylation analysis in those studies.

To effectively boost the power of detecting DMCs/DMRs in DNA methylation studies using low coverage methylation assays, several recent studies have proposed a variety of computational approaches [13–18] for in silico prediction of DNA methylation values at unmeasured CpG sites. Some of these DNA methylation imputation approaches used classical statistical/machine learning methods such as ordinary linear regression, logistic regression, random forest, and support vector machine (SVM) approaches [14, 15], while others have adopted more advanced methods, including functional regression (13), deep learning [16], and gradient boosting [17]. Although these methods have some success in imputing the methylation value, there are also some limitations and weaknesses, especially in the utilization of different kinds of information for imputation. If we assume a data matrix of DNA methylation value with rows representing subjects and columns representing CpG sites, the information used for imputation of missing values of a data matrix can be classified into three categories: (1) external annotation information, (2) correlations between subject/samples (rows), and (3) correlations between neighboring CpG sites (columns). The annotation information used in the existing methods often included co-localization of the CpG sites with DNA sequence annotations (e.g. promoters, gene body, CpG islands), cis-regulatory elements (e.g., DNase I hypersensitive sites, specific transcription factor binding sites, and histone modification marks), as well as DNA sequence properties (e.g. GC content, integrated haplotype scores) [19]. Some imputation methods [14–17] use a large number of annotation information as input features for methylation imputation. However, these features are identical for each subject and thus are not informative for predicting subject-specific DNA methylation patterns. Also, for some tissue/cell types, many of these annotation features are currently not available. Several imputation studies using the correlation between samples as prediction features required measurement of DNA levels available using different platforms or from different sets of samples and established site-specific prediction model only on the overlapped CpG sites in the two different data types. For example, Zhang et al*.* developed a prediction model for predicting methylation level of 450 K-specific probes using the probe shared between the two arrays as predictors, trained and tested on DNA methylation data of tumor tissues from 194 patients measured by both 27 K and 450 K array [13]. Ma et al*.* [18] assumed that locus-specific methylation differences between tissues are highly consistent across individuals and they built linear regression and SVM model to predict locus-specific methylation in the target tissue based on methylation in the surrogate tissue. It remains unclear in those studies if the established model could be applied directly to an external dataset to predict methylation levels. Thus, the practical value of using only the correlation between samples for methylation imputation is vague: if different data types (e.g. DNA methylation data measured in different platforms or tissues) are already available for the same set of subjects, there’s no need for researchers to impute one data type by the other one. Furthermore, the information of neighboring CpGs was highly trivialized in previous methods. For instance, several methods only took an average of the methylation level of one upstream and downstream CpGs weighted by the genomic distance [15], or simply drop those features in the regression model, without taking into account of the whole methylation profile in a genomic region [14]. Fan’s method also assessed the correlation of flanking CpGs in a panel of surrogate tissue and selected the WGBS methylation value from the tissue which has the most similar local methylation pattern as imputation score for the target locus in a target tissue [15], then combined with the weighted sum of methylation levels of the closest CpGs. Although this method integrates the correlation between samples and correlation between neighboring CpGs, it lacks mathematical rigor. Since the local methylation profile is the only subject-specific information among the three kinds of information aforementioned, it should be modeled and integrated with other types of information more carefully to get subject-specific imputation of methylation values. For downstream association analysis in EWAS, only on those CpGs sites with enough variance in methylation values across subjects are informative.

**Fig. 1:**
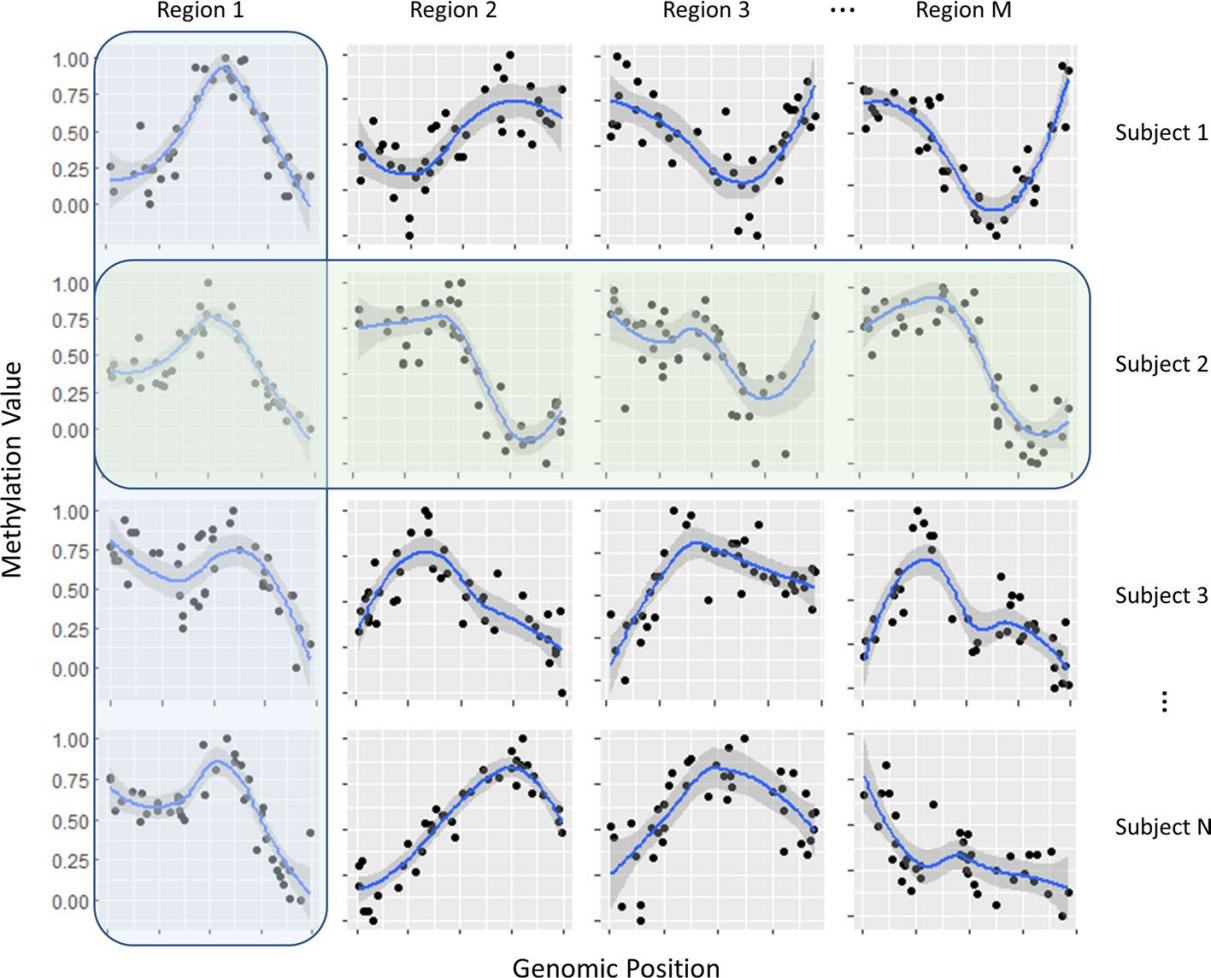
A panel of simulated methylation data to demonstrate our model. The blue box represents the regional model that integrates the similarities in local methylation patterns across subjects, and green box represents the subject model which integrates the similarities in local methylation patterns across multiple genomic regions within a subject

Taking the limitations in the previous methylation imputation methods into account, we developed a novel computational strategy to impute the methylation value at the unmeasured CpG site in the low coverage DNA methylation data. We hypothesized that the linear model of radial basis functions (RBFs) can be used to capture the information of the local methylation profile, and we proposed a mixture regression model (MRM) of RBFs to impute the methylation values in a genomic region for multiple subjects simultaneously. The regional modeling is based on recent findings that DNA methylation has a similar correlation pattern to that of linkage disequilibrium (LD) in genetic SNP variation [20, 21]. Based on the existence of such a correlation structure of neighboring CpG sites in DNA methylation data, the RBFs were used to fit the curve of methylation profile in predefined regions while the MRM can cluster the multiple methylation profiles simultaneously. We fit the MRM in two steps, first across subjects and then across regions (Fig. 1), then ensembled the two imputation values for each missing CpG site with stacked regression. By this approach, the MRM of RBFs can effectively impute the methylation values at the missing CpG sites by taking advantage of the information of neighboring CpGs, the similarities of local methylation patterns not only across subjects but also across multiple genomic regions within each subject.

The rest of the article is organized as follows. In “Results” section, we described the simulation scheme for evaluating the performance of MRM and presented the performance of MRM in both simulated and real methylation data comparing with several other commonly used imputation methods. As an empirical demonstration, we also applied the MRM method to an RRBS methylation dataset for an association study of bone mineral density (BMD). In “Discussion” section, we elaborated on the strength, limitations, and some future extensions of our study. In “Methods” section, we introduced in detail the statistical model of the proposed MRM method.

## Results

To imputing the methylation values, we first built a regional model, which is independent for each predefined genomic region across subjects, assuming similarity in local methylation profiles across subjects. Then we built a subject model in different genomic regions of each subject to get another imputation of the methylation value at the missing points (Fig. 1, details see “Methods”). The final imputation value is the stacked value of imputation values from both the regional and the subject model. The performance of our method was benchmarked on both simulated and empirical data.

### Benchmark the imputation performance on simulated data

We simulated DNA methylation data of multiple regions based on linear models of RBFs. For each region, the subjects were randomly assigned to different clusters. The MRM along with the other four DNA methylation imputation methods were applied to the simulated data, and their performance was evaluated under different conditions with varying noise levels, sample sizes, and missing rates.

### Simulation scheme

#### Simulation by MRM model

To evaluate the imputation performance of the MRM, we simulated methylation of 100 independent regions each with 50 CpG sites. In each region, the methylation values were simulated directly by a mixture regression model of RBFs. Four sub-population clusters were generated with the following cluster proportions: $\pi =$ 10%, 20%, 30%, and 40%. In total, we simulated 12 datasets with varying sample size, noise level, and missing rate to evaluate the performance of MRM in different settings (Additiaonal file 1: Table S1). The sample size was set to be 20, 50, 100, or 200, which is typical in current WGBS studies [22]. To mimic the inherent noise, Gaussian noise with mean zero and varying variance was introduced to the probability of methylated CpG sites. The missing rate, which represents the percentage of CpGs in a region that is not measured, was set to be 20–80%. The wide range of missing rates could represent either the missing rate within the WGBS platform for different subjects or the missing rate in other lower coverage platforms compared with WGBS [11].

####  Simulation by profile-based bisulfite sequencing data simulator

We used the pWGBSSimla software [23] to simulate WGBS data that is more similar to real data. This algorithm calculated the smoothed methylation rates based on the real cell-type-specific methylation profiles. WGBS data in a 100 kb region in chromosome 1 of mesenchymal stem cells of sample size 20 and 100 were simulated, resulting in a total of 850 CpGs per sample. The CpGs were distributed into 17 continuous regions each containing 50 CpGs. We randomly deleted 20–80% CpGs to generate artificial missing values. MRM models were trained by the remaining CpGs to predict the artificial missing values. The center parameter $\mu_{j}$ was set to be equally distributed along the region and the number of RBF centers was set to be 50 for each region. The positions were scaled between − 1 and 1 and the scale parameter $\gamma$ of RBF was set to be − 10.

#### Competing methods

To benchmark the imputation performance of MRM, we compared it with four other imputation strategies that have been proposed and applied in previous studies. First, as a baseline approach, we computed the weighted sum of methylation values of the closest upstream and downstream CpG sites. The weight was inversely proportional to the distance from the target CpG [15]. Using the four features (methylation value and distance of the closest upstream and downstream CpG sites), we trained a random forest (RF) model on each region. This was essentially the same method as proposed in [14], but without adding any external annotation information, in order to ensure that information used for imputation by different methods only comes from methylation data itself so that the comparison was fair. We also applied the k-nearest neighbors (KNN) algorithm, which has been widely used for imputing missing values in gene expression data [24] and also been proposed for imputing DNA methylation data by [14]. As practiced previously [24, 25], the input of the KNN algorithm is a matrix of methylation values with CpG sites in the rows and subjects in the columns. For each CpG site with missing values, the KNN algorithm finds the k nearest neighbors (CpGs) using a Euclidean metric between the columns for which that CpG is not missing and all the columns of each other CpGs in the genomic region, and impute the missing elements by averaging those non-missing elements of its neighbors. If all the neighbors are missing in a particular CpGs, the algorithm will use the regional column mean for that CpG. Finally, we compared our method to a recently developed method, Melissa (MEthyLation Inference for Single cell Analysis), a Bayesian hierarchical method to cluster cells based on local methylation patterns [26]. At each genomic region, Melissa imputation performs clustering for local methylation profiles, which is similar to our model. However, the Melissa model get the cluster membership by clustering the whole methylome of cells instead of clustering in each region independently. As designed for single cell bisulfite sequencing data, the input need to be binary values (0 or 1) indicating the methylation status and the output is a continuous value from 0 to 1. To make the comparion feasible, we binarized the methylation values using cutoff 0.5 to generate the input for Melissa model.

### Effect of noise level

**Fig. 2:**
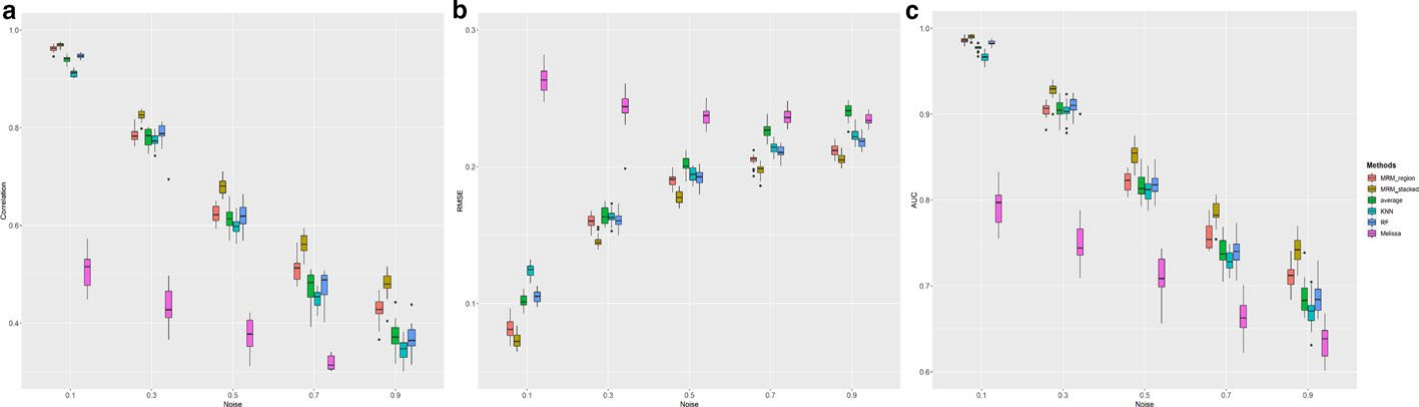
Box plot of **a** Correlation, **b** RMSE, **c** AUC of six imputation algorithm on simulated data under the effect of the noise level. Each colored boxplot indicates the imputation performance on 20 subjects. Abbreviations RF-random forest; KNN-k nearest neighbors; average: one upstream and one downstream weighted sum

To evaluate the robustness of imputation methods to noise, we simulated five datasets by adding different levels of Gaussian noise with varying standard deviations from 0.1 to 0.9. The sample size was set to be 20 and the missing rate to be 20% in all datasets. Figure 2 shows the comparison of different imputation methods with varying noise levels. The performance of all algorithms decreased with the increasing noise level, in terms of significant decrease of correlation, increase of RMSE, and decrease of AUC (test statistics in Additiaonal file 1: Table S2). However, the MRM method consistently outperforms all the other tested methods, and the performance exceedance of MRM over other methods also increases with increasing noise levels.

### Effect of sample size

**Fig. 3:**
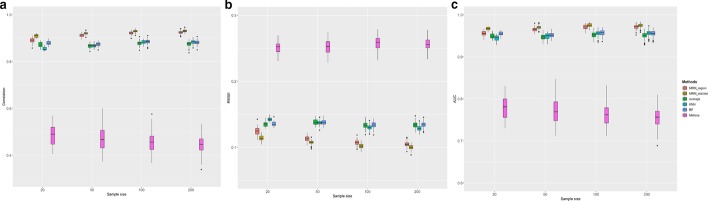
Box plot of **a** correlation, **b** RMSE, **c** AUC of six imputation algorithm on simulated data under the effect of sample size. Each colored boxplot indicates the imputation performance on the given number of subjects. Abbreviations see legend of Fig. 2

Imputation accuracy may be influenced by the sample size of the dataset. A smaller sample size will lead to increases in the variance of imputed values [27]. For algorithms that use many features, the design matrix tends to be ill-conditioned if the sample size is limited. To evaluate the effect of sample size on the performance of our imputation method, we simulated four datasets with sample size 20, 50, 100, and 200. The standard deviation of Gaussian noise was set to be 0.2 and the missing rate was fixed to be 20% at random. As shown in Fig. 3, our method significantly outperformed other methods in terms of correlation, RMSE, and AUC at all sample sizes (test statistics in Additiaonal file 1: Table S3). For most algorithms, the variance in correlation and RMSE are generally increasing with the decrease of sample size. However, the changes of correlation and RMSE of our method with varying sample sizes were less dramatic, highlighting the performance stability of MRM. On the other hand, the performance of RF and Melissa methods even declined with the increasing sample size, suggesting that larger sample size might not always be beneficial for those imputation frameworks that using global features if the methylation data only have regional cluster patterns.

### Effect of data missing rate

**Fig. 4:**
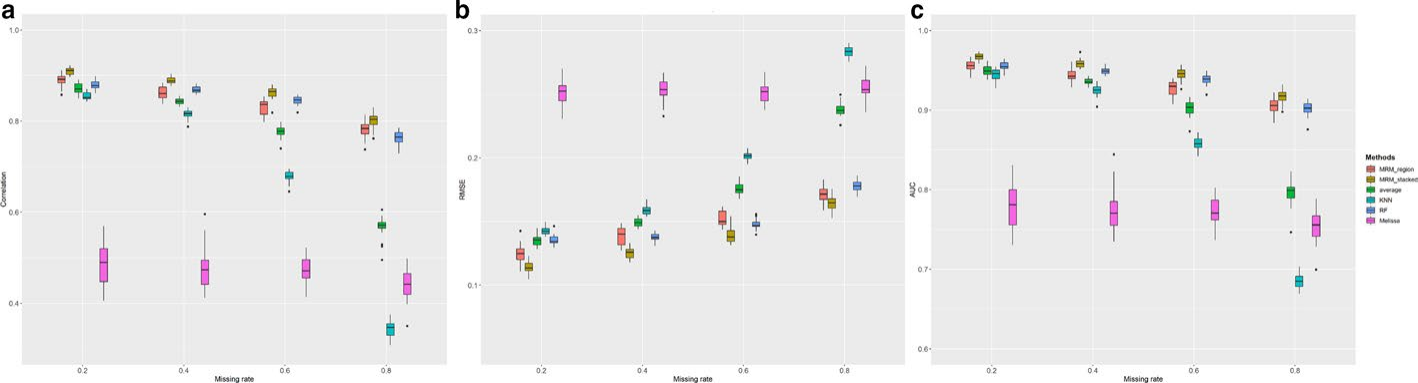
Box plot of **a** correlation, **b** RMSE, **c** AUC of six imputation algorithm on simulated data under the effect of the missing rate. Each colored boxplot indicates the imputation performance on 20 subjects. Abbreviations see legend of Fig. 2

We tested the DNA methylation imputation methods on four simulated data sets with different levels of missing rate (20%, 40%, 60%, and 80%). Like the comparisons for other conditions, our MRM methods significantly outperform other tested methods in terms of correlation, RMSE, and AUC, under each missing rate level (Fig. 4, test statistics in Additiaonal file 1: Table S4). As expected, we observed significant decrease of correlation, increase of RMSE, and decrease of AUC with increasing missing rate for all tested methods, particularly for algorithms using simple local features, e.g. KNN and up/downstream weighted sum algorithm. However, the decrease of imputation performance of our MRM method is much less dramatic than the other methods, demonstrating the exceptional robustness of our methods even when the missing rate is high. This might be due to the utilization of complementary information across subjects, neighboring CpG sites, and other genomic regions.

We also compared the imputation performance on pWGBSSimla simulated data with different levels of missing rate (20%, 40%, 60%, and 80%) at sample size 20 and 100. Although the performance of all methods on pWGBSSimla data was relatively low, the MRM methods still significantly outperform other tested methods under both sample size settings (Additiaonal file 1: Figure S1, Table S5).

### Benchmark the imputation performance on real methylation data

#### Dataset

We downloaded a WGBS dataset measured from subcutaneous adipose tissue (SAT) of 19 subjects in the Multiple Tissue Human Expression Resource (MuTHER) cohort from the ArrayExpress database [access ID: E-MTAB-3549]. The subjects were Caucasian females aged between 40 and 87 years old. The WGBS data measured ~ 27 million CpG sites, with mean genome coverage 6.3-fold (1.0–12.9) and CpG-discovery saturated at sixfold coverage [28].

#### Data preprocess

For demonstration purposes, we only applied the imputation methods to chromosome 18. We clustered the CpG sites on this chromosome into regions based on their physical distance and the similarity of methylation values of two CpG sites by an R package *Aclust* [29]. Specifically, the algorithm first scanned all the CpG sites in WGBS dataset to identify pairs of CpG sites for which the physical distance between the two CpG sites is smaller than 3000 bp and the Pearson correlation of the methylation values at the two sites is greater than 0.3, then the two CpG sites with all the sites wedged in between them were merged into one region. Only two adjacent regions can be merged at each iterative step. The similarity of methylation values in two regions was defined using the average correlation between all sites in the two regions.

We mapped the CpG sites to promoter regions of up to $\pm$ 5 kb around the transcription start site (TSS) of UCSC genes. The promoter region containing more than 50 measured CpG sites were then filtered by variance, skewness [30] and runs test for non-randomness [31]. The promoter region was kept for imputation only if the methylation data in that region was available for more than 15 subjects, with variance > 0.1, skewness > − 1, and the number of runs < 15. As many CpG sites are either completely methylated or unmethylated across individuals in a genomic region [18] the filtering criteria were applied to make sure the pattern of methylation profile in the selected region follows the assumption of the mixture of regression models. After applying the clustering and filtering, a total of 47 regions with more than 50 CpGs were selected. And we define this scenario as *Condition 0*. To imitate the conditions of various missing rates, we randomly deleted the measured DNA methylation values at 20%, 40%, 60%, and 80% of CpG sites in the selected regions of each subject to generate artificial missing values.

While the clustering and filtering procedures aimed to ensure the existence of LD-like patterns [20] in the selected genomic regions and to make the assumptions of MRM satisfied, we further investigate the performance of MRM in the following two more general conditions in which the assumption of MRM might be violated: *Condition 1* We randomly selected 2500 continuous CpGs without clustering or filtering and divided them into 50 sliding windows with 50 CpG; *Condition 2* We randomly selected 50 promoter regions and pruned them to regions that contain 50 CpGs (25 CpGs upstream and 25 CpGs downstream the TSS), without performing clustering and filtering.

#### Imputation

After generating the artificial missing values, the methylation data of the remaining CpGs were used to train the MRMs. The center parameter $\mu_{j}$ and scale parameter of the RBFs was set to be the same as the pWGBSSimla simulated data (see “Simulation by profile-based bisulfite sequencing data simulator” in “Result”). We applied our proposed method to impute the missing values at the target CpG sites based on the regional model only and the stacked model (weighted sum of the regional and the subject model). The other four methods were also used to impute these missing values and then compared with our methods under different settings of missing rates.

#### Performance on real data

**Fig. 5:**
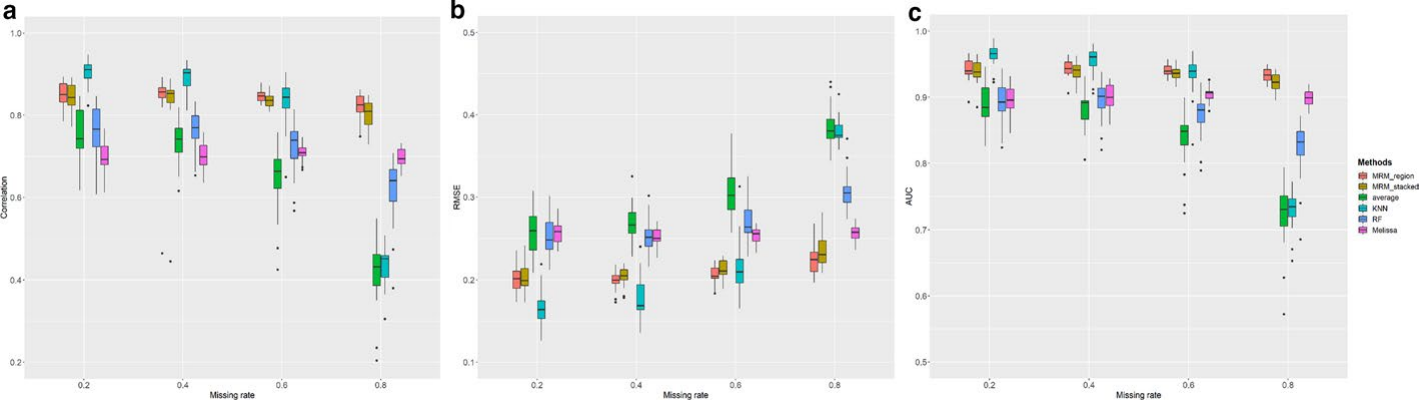
Box plot of **a** correlation, **b** RMSE, **c** AUC of six imputation algorithms on real WGBS data under the effect of the missing rate. Each colored boxplot indicates the imputation performance on 19 subjects. Abbreviations see legend of Fig. 2

The comparison of correlation, RMSE, and AUC between our methods and other methods for imputing the DNA methylation values at the targeted CpG sites were shown in Fig. 5. Consistent with the simulation results, we observed decreased correlation, increased RMSE, and decreased AUC with an increasing missing rate for all methods. At the lower missing rate (r = 0.2 and 0.4), MRM exhibited comparable performance with KNN and outperforms all the other tested imputation methods. However, the correlation drops dramatically for KNN and up/downstream weighted sum methods, similar to the simulation results. MRM also outperformed other methods at higher missing rates (r = 0.6 and 0.8), highlighting the strength of MRM for taking advantage of information from multiple sources. It is noteworthy that the Melissa algorithm performs relatively stable under the influence of the missing rate compared with other methods. Additionally, MRM using only the regional model performs slightly better than using the stacked regional and subject model, indicating that the local methylation patterns across multiple genomic regions within a subject introduced more noise than useful information for methylation imputation. The performance of MRM under *conditions 1 and 2* is similar to that under *condition 0* (Additiaonal file 1: Figures S2, S3). However, the performance of MRM at the same missing rate across different conditions are significantly different (Additiaonal file 1: Table S6). The MRM achieves the best performance in regions selected with clustering and filtering (*condition 0*) among the three conditions. Although its performance in regions defined by the sliding window approach (*condition 1*), which ignored the correlation pattern in methylation data, is inferior to the other two conditions, the MRM methods still outperformed other methods at a high missing rate. This result also emphasized that the regional correlation pattern is important for MRM to make accurate imputation.

### Application to an epigenomic association study

After benchmarking the imputation accuracy of MRM, we further demonstrated the empirical implementation of our method by applying the MRM to an RRBS dataset from our recent epigenomic association study of BMD [32]. In this study, DNA methylation profiles of peripheral blood monocytes (PBM) were determined by RRBS in 118 Caucasian females, including 64 subjects with high hip BMD (Z-scores ≥ 0.8) and 54 subjects with low hip BMD (Z-scores ≤ − 0.8). The BMD Z-score was defined as the number of standard deviations a subject’s BMD differed from the mean BMD of their age-, gender-, and ethnicity-matched population. We are particularly interested in imputing methylation values on chromosome 17 because recent studies have found several genes on chromosome 17 were associated with BMD [33] and the methylation level of a set of CpGs on chromosome 17 mediated the association between SNP and BMD [34]. MRM was applied to promoter regions ($\pm$ 5 kb around TSS) where more than 50 CpGs were measured in each region taking all the subjects into account. To increase the computational speed, only the regional model is applied. We used *methylKit* [35] to identify DMCs between low BMD and high BMD group, adjusting for age, body mass index (BMI), drinking status, smoking status, and 1st principal component (PC) of methylation. Before imputation, only CpG sites with ≥ threefold coverage in at least 30 subjects in each BMD group were included in the association analysis, while after imputation, all the CpG sites in the processed promoter regions were included. CpGs with significant difference in methylation levels (Bonferroni adjusted p-value < 0.05) between the two BMD groups were defined as DMCs. The DMCs were annotated to the genes corresponding to the promoter region. The Genomic Regions Enrichment of Annotations Tool (GREAT) v3.0.0 [36] was used to evaluate whether the nearby genes of DMCs are enriched in any gene and human phenotypes ontology terms.

We limited our analyses to 35,713 CpGs in 1,369 qualified promoter regions on chromosome 17 for demonstration purposes. Without imputation, we only identified 348 DMCs with p-value < 1.49E−6 (0.05/35,713) significantly associated with BMD. In contrast, by applying the MRM algorithm, we imputed methylation values for 309,165 CpGs in the tested promoter regions and identified 2459 significant DMCs with p-value < 1.62E−7 (0.05/309,165), among which 2452 were new DMCs. The DMCs were distributed in 594 unique promoter regions (median number of DMCs in a promoter region = 2). The GREAT analysis showed the nearby genes of the imputed DMCs were significantly enriched in some BMD related terms of mouse phenotype (Table 1). We further checked the functions of the 42 genes whose promoter regions contained more than 10 imputed DMCs. Interestingly, six genes (*SPECC1, SPATA20, USP36, SMG6, MYO1D*, and *ASB16-AS1*), have been found to be associated with BMD or bone-related phenotypes in previous studies [37–43] (Table 2, Additiaonal file 1: Table S7). These results indicated that our algorithm can successfully impute the methylation values at the unmeasured CpG sites and enhance the power to identify novel DMCs in epigenomic association studies.

**Table 1:** Mouse phenotype enrichments for BMD-associated DMCs

Term name	Fold enrichment	FDR Q-Val
Brachyphalangia	21.48521	3.95E−33
Short metacarpal bones	21.48521	3.95E−33
Short metatarsal bones	21.48521	3.95E−33
Decreased trabecular bone mass	21.48521	3.95E−33
Decreased trabecular bone connectivity density	9.427838	3.95E−21

**Table 2:** DMC enriched genes that were associated with BMD or bone metabolism

Gene	Number of imputed DMCs	Traits	GwasCatlog Study ID	PMID
SPECC1	16	Heel BMD	GCST006433	30048462
SPATA20	23	Heel BMD	GCST007066	30595370
USP36	11	Heel BMD	GCST006979	30598549
SMG6	13	Heel BMD	GCST006979	30598549
		Heel BMD	GCST006433	30048462
MYO1D	13	Osteoclast differentiation	NA	21567867
ASB16-AS1	12	BMD	NA	29763751

## Discussion

We proposed a novel computational strategy, MRM of RBFs, to impute the missing or unmeasured CpG methylation values by effectively integrating three kinds of information: the information of neighboring CpGs, the similarities in local methylation patterns across subjects, and the similarities in local methylation patterns across multiple genomic regions within a subject, thus addressed the low coverage problem of the methylation data generated in many cost-efficient platforms. The MRM method can be used to impute methylation values in pre-defined genomic regions, for example, the promoter region or to impute methylation values on the genome-wide scale using sliding windows. The real data benchmarking results of MRM performance under several preprocessing conditions indicated that this method is more suitable for imputing missing values on a regional basis, especially when the regions have an LD-like correlation pattern. In this study, we only implement the MRM on selected genome regions for demonstration purposes. The computation time of MRM is practicable for implementing on a whole genome scale, although the computation time is longer compared with other methods under different sample sizes for imputing simulated data (Additiaonal file 1: Table S8). Considering the excellence in performance at a high missing rate, it worth trading a reasonable increase amount of computation time for the imputation accuracy. The MRM imputation does not require any external information, such as regulatory annotation and DNA sequence properties. It is straight forward to fit the output of MRM as a new local feature into some existing methylation imputation frameworks as [15] and [14].

Based on our simulation result, the MRM method could recover the WGBS data (correlation = 0.8) after deleting 80% of data points. This missing rate could represent the difference in genome coverage between WBGS and RRBS. Thus, our method could be applied to most of the existing RRBS data to expand the coverage and achieve better imputation accuracy. However, genome coverage of microarray-based methods are even lower, analogous to WGBS data with a missing rate > 97%. It will be unrealistic to recover whole-genome methylation data with that high missing rate without reference or external information. We would suggest not use MRM to impute microarray data only. If WGBS data from the same cell type were available, users could combine methylation data from different platforms (i.e. WGBS and microarray) as input, and the higher coverage methylation data will perform as a reference for imputation.

Despite the advantages of the MRM algorithm, we noticed that it is important to evaluate the discrepancies between the real methylation data and the statistical model we proposed. As opposed to the simulated data, the subject model provided more noise than useful information in the real data and thus the stacked model performed less well than the regional model. This may due to the fact that the selected regions in our study do not share common patterns across the genome, which conflicts assumption of the subject MRM model. We believe that common patterns exist across certain genome regions, e.g. promoters of genes in the same pathway or regulatory network. To ensure the stacked model works better, the users may need to incorporate some prior annotation information in selecting specific regions they want to impute. Otherwise, we recommend using the regional model in general.

Some other future directions of the MRM algorithm are worth exploring. First, since MRM is a finite mixture regression model, the number of clusters has to be specified. It is computationally burdensome to fit multiple MRMs and do model selection based on the model likelihood. Instead of doing the model selection from several models with different cluster number, we recommend users choose a reasonably larger cluster number. As we found in the simulation study that when the number of clusters is set to be larger than the real number of clusters, the performance of MRM as good as if the number of clusters is correctly specified (Additiaonal file 1: Figure S4). An alternative scheme would assume a mixture model with an unknown number of components and the most common choice of the prior distributions for this clustering problem is the Dirichlet process (DP) [44]. The DP mixture model has been successfully used to perform clustering of gene expression data by microarray [45, 46], and could be extended to DNA methylation analysis in future studies. Besides, the association analysis after imputation is conducted throughout a large number of CpG sites, which will lead to severe multiple testing problems. It has been well recognized that DMRs might have more prominent biological significance compared with single CpG sites [47]. Therefore, we will attempt to integrate the MRM imputation algorithm with region-based differential methylation analysis approaches [21, 48–50] to develop novel computation tools that could simultaneously do DNA methylation imputation and region-based association testing.

## Conclusions

The proposed MRM method provided a state-of-art performance for methylation data imputation. On both simulated and empirical DNA methylation data, the MRM method achieved a better imputation performance over a set of competing methods, particularly when the missing rate is high. By applying the proposed method to an in-house DNA methylation for osteoporosis, we identified some novel differential methylation signals that are significantly associated with BMD and demonstrated that this method is well applicable to existing methylation studies that were conducted with commonly used, low genome coverage methylation analysis platforms and is expected to significantly enhance the discovery of novel DNA methylation regulated genes and mechanisms underlying various human disorders/traits.

## Methods

### Statistical modeling of the methylation profile

As in many previous practical studies, we are interested in imputing the methylation level of a CpG site as a ratio ranging from 0 to 1, where 0 represents no methylation and 1 represents 100% methylation at a CpG site. We assumed that the methylation data of the $m$-th genomic region in the $n$-th subject is represented by a vector $y_{mn}$ of length $I$, where ${\text{m}} = 1,2...,{\text{M}}$ and ${\text{n}} = 1,2,...,{\text{N}}$.

#### Regional model

A subject-specific model was developed for methylation profiles in each predefined region. For each region $m$, we assumed that the methylation profiles of the $N$ subjects can be partitioned into at most $K$ clusters. Suppose there are $I$ measured CpG sites. For one subject, let ${\varvec{x}} = \left( {x_{1} ,x_{2} , \ldots x_{I} } \right)$ be a vector of the CpG locations;$f\left( {\varvec{x}} \right)$ be a function representing the methylation profile. We assumed $f\left( {\varvec{x}} \right)$ is a linear combination of a set of radial basis functions $h_{j} \left( \right)$ of the input space ${\varvec{x}}$

1 $${\text{y}} = { }f\left( {{\varvec{x}},{\varvec{w}}} \right) = { }\mathop \sum \nolimits_{j = 0}^{J} w_{j} h_{j} \left( {\varvec{x}} \right) = H{\varvec{w}}$$

where $H$ is an $I \times J$ design matrix with element $h_{ij} = \exp \left( { - \delta \left| {\left| {x_{i} - \mu_{j} } \right|} \right|^{2} } \right)$; $x_{i}$ are the component of vector $\user2{ x}$; $\mu_{j}$ are the selected centers of the basis functions; $\delta$ is the scale parameter; ${\varvec{w}} = \left( { w_{1} , \ldots , w_{J} } \right)^{T}$; $\mu_{j}$ represents the RBF centers; $J$ represents the number of RBF centers.

Parameters ${\varvec{w}}$ in model [1] can be obtained simply by solving the normal equation

$$\left( {H^{\prime}H} \right)\hat{\user2{w}} = H{^{\prime}}{\varvec{y}}$$

For $N$ subjects from $K$ clusters i.e. regression with $K$ specific set of regression coefficients, the probability that subject $n$ belong to cluster $k$ is $\pi_{k}$. The methylation profile of subject $n$ region $m$ can be written as

2 $$y_{in} = { }f\left( {{\varvec{x}},{\varvec{w}}} \right) = { }\mathop \sum \nolimits_{j = 0}^{J} w_{kjmn} {\text{exp}}\left( { - \delta \left| {\left| {x_{i} - \mu_{j} } \right|} \right|^{2} } \right){ }\;{\text{if}}\quad z_{kn} = 1$$

where $z_{nk}$ is a latent indicator variable of whether subject $n$ belongs to cluster $k$, $z_{nk} \in \left\{ {0,1} \right\}$ and $p\left( {z_{kn} = 1} \right) = \pi_{k}$.

The observed methylation data $\left( {{\varvec{x}},{\varvec{y}}} \right)$ can be viewed as data repeatedly measured from $N$ subjects, each has $I$ observations. Thus, the conditional log-likelihood of $Y$ can be written as a weighted sum of $K$ Gaussian distributions.

3 $$L({\varvec{y}}|{\varvec{x}}) = \mathop \sum \nolimits_{n = 1}^{N} \mathop \sum \nolimits_{i = 1}^{I} log\mathop \sum \nolimits_{k = 1}^{K} \pi_{k} N(H\left( {x_{in} )w_{k} {|} \Sigma } \right)$$

To impute the missing values, we first need to learn the model parameters of each cluster and the posterior probability that a subject belongs to cluster $k$ using the available data in a genomic region of all subjects. Then, given the genomic position of the missing point, the methylation value can be computed based on Eq. (2). The regression coefficient $w_{k}$ can be learned by maximizing Eq. (3). But the $H$ matrix could become high dimensional as the number of RBFs increases and the number of observed CpG sites remains fixed. To ameliorate this issue, we maximize a penalized version of Eq. (3), by adding an $l1$ regularization term to the log-likelihood function which will encourage the weights to decay to zero as proposed by Stadler [51].

4 $$L_{pen} \left( {y|x} \right) = \mathop \sum \nolimits_{n = 1}^{N} \mathop \sum \nolimits_{i = 1}^{I} log\mathop \sum \nolimits_{k = 1}^{K} \pi_{k} N(H\left( {x_{in} )w_{k} {|} \Sigma } \right){ } + {\uplambda }\mathop \sum \nolimits_{k = 1}^{K} \pi_{k} \left| {\left| {w_{k} } \right|} \right|_{1}$$

The parameter set $\theta = \left\{ {\pi_{k} , {\varvec{w}}_{{\varvec{k}}} ,\user2{ }{\Sigma }} \right\}$ was estimated by maximizing the penalized likelihood function using the EM algorithm using functions in R package *FlexMix* [52].

Since we don’t know the true clustering of subjects, we fit the MRMs with different numbers of clusters ($K$). The maximum number is set to be proportional to the sample size $N$. The model with K clusters that yields the lowest ICL (Integrated completed likelihood) was retained, which has been proven to be a very popular approach to choose the number of clusters in model-based clustering [53]. The penalty parameter $\lambda$ is chosen in grid search by a tenfold cross validation.

### Subject model and stacking

While the regional model assumes similarity in local methylation profiles across subjects, some studies also found that the methylation profile in proximal regions with similar annotation properties may share the same patterns [13, 14]. Thus, we fit the MRM model in different genomic regions of each subject to get another imputation of the methylation value at the missing points (Fig. 1). The model fitting process is analogous to the regional model.

To integrate multiple imputation models, we used a least-square regression model to combine the outputs from the regional and subject models. This stacking approach forms linear combinations of different predictors at each locus to improve the prediction accuracy [54].

5 $${\varvec{\beta}} = argmin \left[ {y - \left( {\beta_{r} \widehat{{y_{r} }} + \beta_{s} \widehat{{y_{s} }}} \right)} \right] s.t.\beta_{r} ,\beta_{s} > 0$$

where $\widehat{{y_{r} }}$ and $\widehat{{y_{s} }}$ are imputed methylation values from the regional and the subject models, respectively. The non-negative regularization has been shown to produce stacking predictors with substantially reduced prediction errors [54]. To train the stacking regression model, we adopted bootstrapping to randomly generate artificial missing values at the locations which were not overlapped with true missing locations. The weights were estimated by Eq. (5) based on the imputed and true values on the artificial missing points. The averaged value of each weight on 100 times bootstrapping was used for prediction.

### Evaluation metric

The performance was evaluated by the root mean squared error (RMSE) and the correlation between the true and imputed values, which are defined as the following:

$$RMSE = \sqrt {\frac{{\mathop \sum \nolimits_{m = 1}^{M} \mathop \sum \nolimits_{i = 1}^{{I_{m} }} \left( {y_{im} - \widehat{{y_{im } }}} \right)^{2} }}{MI}}$$

$$correlation = \frac{{\mathop \sum \nolimits_{m = 1}^{M} \mathop \sum \nolimits_{i = 1}^{{I_{m} }} \left( {y_{im} - \overline{{y_{im} }} } \right)\left( {\widehat{{y_{im} }} - \overline{{\widehat{{y_{im} }}}} } \right)}}{{\sqrt {\mathop \sum \nolimits_{m = 1}^{M} \mathop \sum \nolimits_{i = 1}^{{I_{m} }} \left( {y_{im} - \overline{{y_{im} }} } \right)^{2} } \sqrt {\mathop \sum \nolimits_{m = 1}^{M} \mathop \sum \nolimits_{i = 1}^{{I_{m} }} \left( {\widehat{{y_{im} }} - \overline{{\widehat{{y_{im} }}}} } \right)^{2} } }}$$

where $y_{im}$ and $\widehat{{y_{im} }}$ are the true and imputed value of the $i$-th missing point in the $m$-th region of the genome in a subject, respectively, $I_{m}$ is the number of missing points in region $m$ and $M$ is the number of genomic regions. In addition, to make fair comparisons with Melissa which only takes binary methylation values as input, the area under the receiver operating characteristic curve (AUC) of the true and predicted values with a cut-off of 0.5 was also calculated for all the tested imputation methods. We performed t-tests to compare the AUC, correlation, and RMSE under different settings to determine whether observed differences between the performance were statistically significant.

## Supplementary information

**Additional file 1.** Supplementary materials (Supplementary Tables S1–S8, Supplementary Figures S1–S4).

## Data Availability

The source code is available at https://github.com/yuft2003/MRM.The WGBS datasets for benchmarking are available in the ArrayExpress database (access ID: E-MTAB-3549) and RRBS datasets of osteoporosis are available in dbGaP database (access ID: phs001960.v1.p1).

## Abbreviations

AUC: Area under the receiver operating characteristic curve
BMD: Bone mineral density
CpG: Cytosine and guanine dinucleotides
DMC: Differentially methylated CpG
DMR: Differentially methylated region
DP: Dirichlet process
ESCs: Embryonic stem cells
EWAS: Epigenome-wide association study
GREAT: Genomic Regions Enrichment of Annotations Tool
KNN: K-nearest neighbors
LD: Linkage disequilibrium
Melissa: Methylation inference for single-cell analysis
MRM: Mixture of regression models
MUTHER: Multiple Tissue Human Expression Resource
RBF: Radial basis functions
RF: Random forest
RMS: Eroot mean squared error
RRBS: Reduced representation bisulfite sequencing
SAT: Subcutaneous adipose tissue
SVM: Support vector machine
TSS: Transcription start site
WGBS: Whole-genome bisulfite sequencing
