# Supplementary material for: A novel computational strategy for DNA methylation imputation using mixture regression model (MRM)
Source: BMC Bioinformatics. 2020 Dec 1;21:552. doi: 10.1186/s12859-020-03865-z (PMC7708217; doi:10.1186/s12859-020-03865-z)
Supplement: Supplementary file 1 — Additional file 1. Supplementary materials (Supplementary Tables S1–S8, Supplementary Figures S1–S4). [file 12859_2020_3865_MOESM1_ESM.docx]

Supplementary Materials of

A Novel Computational Strategy for DNA Methylation Imputation Using Mixture Regression Model (MRM)

Fangtang Yu, Chao Xu, Hong-Wen Deng, Hui Shen

# Tables

## Table S1. Simulation parameters

| simNo | N | noise | missingRate | k.region.max |
| --- | --- | --- | --- | --- |
| 1 | 20 | 0.1 | 0.2 | 6 |
| 2 | 20 | 0.2 | 0.2 | 6 |
| 3 | 20 | 0.3 | 0.2 | 6 |
| 4 | 20 | 0.5 | 0.2 | 6 |
| 5 | 20 | 0.7 | 0.2 | 6 |
| 6 | 20 | 0.9 | 0.2 | 6 |
| 7 | 20 | 0.2 | 0.4 | 6 |
| 8 | 20 | 0.2 | 0.6 | 6 |
| 9 | 20 | 0.2 | 0.8 | 6 |
| 10 | 50 | 0.2 | 0.2 | 6 |
| 11 | 100 | 0.2 | 0.2 | 8 |
| 12 | 200 | 0.2 | 0.2 | 10 |

## Table S2a. Test of difference in correlation under different noise level

| *variance of Gaussian noise = 0.1* | | | |  |  |
| --- | --- | --- | --- | --- | --- |
|  | median | mean | sd | t | p.value |
| cor_MRM_region | 0.962786 | 0.961954 | 0.006595 | NA | NA |
| cor_MRM_stacked | 0.970083 | 0.969804 | 0.003972 | -4.56007 | 7.46E-05 |
| cor_average | 0.941495 | 0.939902 | 0.006839 | 10.37953 | 1.22E-12 |
| cor_KNN | 0.912237 | 0.911195 | 0.006401 | 24.69928 | 5.38E-25 |
| cor_RF | 0.946837 | 0.94677 | 0.004702 | 8.383044 | 8.01E-10 |
| cor_Melissa | 0.515446 | 0.509419 | 0.037731 | 52.8358 | 4.26E-23 |
| *variance of Gaussian noise = 0.3* | | | |  |  |
|  | median | mean | sd | t | p.value |
| cor_MRM_region | 0.782725 | 0.784612 | 0.014055 | NA | NA |
| cor_MRM_stacked | 0.82615 | 0.82537 | 0.010727 | -10.3093 | 3.19E-12 |
| cor_average | 0.783986 | 0.780438 | 0.018573 | 0.801342 | 0.428282 |
| cor_KNN | 0.772531 | 0.773429 | 0.015586 | 2.382762 | 0.022345 |
| cor_RF | 0.787987 | 0.789786 | 0.017744 | -1.02228 | 0.313447 |
| cor_Melissa | 0.427516 | 0.443264 | 0.06925 | 21.60348 | 1.30E-15 |
| *variance of Gaussian noise = 0.5* | | | |  |  |
|  | median | mean | sd | t | p.value |
| cor_MRM_region | 0.621692 | 0.622641 | 0.017377 | NA | NA |
| cor_MRM_stacked | 0.68049 | 0.680886 | 0.01716 | -10.6659 | 5.53E-13 |
| cor_average | 0.613368 | 0.611854 | 0.022743 | 1.685448 | 0.100663 |
| cor_KNN | 0.597806 | 0.598002 | 0.017795 | 4.430305 | 7.73E-05 |
| cor_RF | 0.61892 | 0.61706 | 0.02349 | 0.854196 | 0.398805 |
| cor_Melissa | 0.363327 | 0.363823 | 0.043485 | 24.71735 | 4.78E-19 |
| *variance of Gaussian noise = 0.7* | | | |  |  |
|  | median | mean | sd | t | p.value |
| cor_MRM_region | 0.512918 | 0.510929 | 0.02565 | NA | NA |
| cor_MRM_stacked | 0.561323 | 0.562227 | 0.023009 | -6.65767 | 7.58E-08 |
| cor_average | 0.482602 | 0.473193 | 0.031065 | 4.189113 | 0.000168 |
| cor_KNN | 0.45417 | 0.449605 | 0.016979 | 8.915591 | 2.67E-10 |
| cor_RF | 0.488358 | 0.474657 | 0.030604 | 4.062219 | 0.000244 |
| cor_Melissa | 0.283931 | 0.284815 | 0.031106 | 25.08091 | 1.11E-24 |
| *variance of Gaussian noise = 0.9* | | | |  |  |
|  | median | mean | sd | t | p.value |
| cor_MRM_region | 0.42785 | 0.427178 | 0.025741 | NA | NA |
| cor_MRM_stacked | 0.480039 | 0.479343 | 0.026179 | -6.35417 | 1.86E-07 |
| cor_average | 0.371191 | 0.373344 | 0.02779 | 6.355593 | 1.90E-07 |
| cor_KNN | 0.346239 | 0.341679 | 0.026627 | 10.32416 | 1.42E-12 |
| cor_RF | 0.36427 | 0.368418 | 0.027566 | 6.967375 | 2.79E-08 |
| cor_Melissa | 0.240769 | 0.234644 | 0.034286 | 20.083 | 6.97E-21 |
| *t: t-test statistic compared with MRM_region in each setting* | | | | | |

## Table S2b. Test of difference in RMSE under different noise level

| *variance of Gaussian noise = 0.1* | | | |  |  |
| --- | --- | --- | --- | --- | --- |
|  | median | mean | sd | t | p.value |
| rmse_MRM_region | 0.081123 | 0.081303 | 0.007188 | 0 | 1 |
| rmse_MRM_stacked | 0.07236 | 0.072788 | 0.004974 | 4.356758 | 0.000116 |
| rmse_average | 0.101215 | 0.101781 | 0.005351 | -10.2198 | 4.61E-12 |
| rmse_KNN | 0.124792 | 0.124429 | 0.004432 | -22.8395 | 3.21E-21 |
| rmse_RF | 0.105259 | 0.105032 | 0.004313 | -12.6603 | 8.22E-14 |
| rmse_Melissa | 0.263385 | 0.263917 | 0.010095 | -65.902 | 9.87E-38 |
| *variance of Gaussian noise = 0.3* | | | |  |  |
|  | median | mean | sd | t | p.value |
| rmse_MRM_region | 0.160345 | 0.159579 | 0.005398 | 0 | 1 |
| rmse_MRM_stacked | 0.144493 | 0.14562 | 0.004409 | 8.956708 | 9.46E-11 |
| rmse_average | 0.163368 | 0.164504 | 0.006196 | -2.68016 | 0.010891 |
| rmse_KNN | 0.162919 | 0.163257 | 0.004909 | -2.25446 | 0.030067 |
| rmse_RF | 0.160439 | 0.161089 | 0.00569 | -0.86106 | 0.394623 |
| rmse_Melissa | 0.243977 | 0.243161 | 0.013025 | -26.5109 | 5.31E-20 |
| *variance of Gaussian noise = 0.5* | | | |  |  |
|  | median | mean | sd | t | p.value |
| rmse_MRM_region | 0.190775 | 0.189843 | 0.004404 | 0 | 1 |
| rmse_MRM_stacked | 0.177598 | 0.177872 | 0.00501 | 8.025602 | 1.19E-09 |
| rmse_average | 0.200455 | 0.201635 | 0.006064 | -7.0369 | 3.60E-08 |
| rmse_KNN | 0.194387 | 0.194473 | 0.005028 | -3.09786 | 0.003691 |
| rmse_RF | 0.192561 | 0.19245 | 0.006057 | -1.55689 | 0.128571 |
| rmse_Melissa | 0.237394 | 0.236465 | 0.006674 | -26.0735 | 1.49E-23 |
| *variance of Gaussian noise = 0.7* | | | |  |  |
|  | median | mean | sd | t | p.value |
| rmse_MRM_region | 0.205848 | 0.204784 | 0.00445 | 0 | 1 |
| rmse_MRM_stacked | 0.198486 | 0.197011 | 0.004336 | 5.594764 | 2.05E-06 |
| rmse_average | 0.226766 | 0.226416 | 0.005994 | -12.9584 | 6.24E-15 |
| rmse_KNN | 0.214226 | 0.213668 | 0.004323 | -6.40369 | 1.60E-07 |
| rmse_RF | 0.210435 | 0.210575 | 0.004723 | -3.99088 | 0.000291 |
| rmse_Melissa | 0.235912 | 0.236084 | 0.005502 | -19.7815 | 4.56E-21 |
| *variance of Gaussian noise = 0.9* | | | |  |  |
|  | median | mean | sd | t | p.value |
| rmse_MRM_region | 0.21185 | 0.212066 | 0.004377 | 0 | 1 |
| rmse_MRM_stacked | 0.204939 | 0.20576 | 0.004283 | 4.604791 | 4.53E-05 |
| rmse_average | 0.240958 | 0.240307 | 0.005763 | -17.4518 | 5.54E-19 |
| rmse_KNN | 0.221936 | 0.222709 | 0.005463 | -6.79962 | 5.80E-08 |
| rmse_RF | 0.218756 | 0.218294 | 0.004318 | -4.52975 | 5.70E-05 |
| rmse_Melissa | 0.233819 | 0.234375 | 0.004285 | -16.2883 | 1.00E-18 |
| *t: t-test statistic compared with MRM_region in each setting* | | | | | |

## Table S2c. Test of difference in AUC under different noise level

| *variance of Gaussian noise = 0.1* | | | |  |  |
| --- | --- | --- | --- | --- | --- |
|  | median | mean | sd | t | p.value |
| auc_MRM_region | 0.986157 | 0.986047 | 0.003811 | 0 | 1 |
| auc_MRM_stacked | 0.990577 | 0.989978 | 0.002622 | -3.80047 | 0.000577 |
| auc_average | 0.977747 | 0.977245 | 0.003749 | 7.364051 | 7.97E-09 |
| auc_KNN | 0.966664 | 0.966325 | 0.005638 | 12.96029 | 1.42E-14 |
| auc_RF | 0.98242 | 0.982946 | 0.003075 | 2.831923 | 0.007496 |
| auc_Melissa | 0.797054 | 0.792954 | 0.022916 | 37.17282 | 5.71E-20 |
| *variance of Gaussian noise = 0.3* | | | |  |  |
|  | median | mean | sd | t | p.value |
| auc_MRM_region | 0.906633 | 0.905097 | 0.008351 | 0 | 1 |
| auc_MRM_stacked | 0.929444 | 0.927778 | 0.008462 | -8.53176 | 2.32E-10 |
| auc_average | 0.904533 | 0.906165 | 0.009943 | -0.36774 | 0.715166 |
| auc_KNN | 0.903029 | 0.903654 | 0.011022 | 0.466743 | 0.643543 |
| auc_RF | 0.910129 | 0.910474 | 0.009306 | -1.92313 | 0.062068 |
| auc_Melissa | 0.74399 | 0.75453 | 0.040425 | 16.31237 | 2.94E-13 |
| *variance of Gaussian noise = 0.5* | | | |  |  |
|  | median | mean | sd | t | p.value |
| auc_MRM_region | 0.822881 | 0.821292 | 0.011276 | 0 | 1 |
| auc_MRM_stacked | 0.854666 | 0.852138 | 0.012115 | -8.33473 | 4.34E-10 |
| auc_average | 0.813201 | 0.816085 | 0.015796 | 1.199754 | 0.238439 |
| auc_KNN | 0.81227 | 0.811731 | 0.013418 | 2.439537 | 0.019633 |
| auc_RF | 0.817763 | 0.818383 | 0.015071 | 0.690981 | 0.49411 |
| auc_Melissa | 0.708472 | 0.708868 | 0.025223 | 18.19738 | 1.99E-16 |
| *variance of Gaussian noise = 0.7* | | | |  |  |
|  | median | mean | sd | t | p.value |
| auc_MRM_region | 0.754039 | 0.75705 | 0.014825 | 0 | 1 |
| auc_MRM_stacked | 0.782331 | 0.78493 | 0.013381 | -6.24333 | 2.75E-07 |
| auc_average | 0.737283 | 0.74024 | 0.018314 | 3.190443 | 0.002921 |
| auc_KNN | 0.727876 | 0.728912 | 0.012488 | 6.491827 | 1.37E-07 |
| auc_RF | 0.740049 | 0.738986 | 0.017605 | 3.510011 | 0.001199 |
| auc_Melissa | 0.662589 | 0.662809 | 0.019789 | 17.04494 | 1.37E-18 |
| *variance of Gaussian noise = 0.9* | | | |  |  |
|  | median | mean | sd | t | p.value |
| auc_MRM_region | 0.712032 | 0.71083 | 0.014582 | 0 | 1 |
| auc_MRM_stacked | 0.742034 | 0.740802 | 0.01714 | -5.95625 | 7.16E-07 |
| auc_average | 0.68307 | 0.687142 | 0.018929 | 4.433433 | 8.48E-05 |
| auc_KNN | 0.670757 | 0.671117 | 0.018362 | 7.574361 | 5.73E-09 |
| auc_RF | 0.684154 | 0.684938 | 0.01646 | 5.265723 | 5.99E-06 |
| auc_Melissa | 0.636814 | 0.633097 | 0.020236 | 13.93747 | 9.59E-16 |
| *t: t-test statistic compared with MRM_region in each setting* | | | | | |

## Table S3a. Test of difference in correlation under different sample size

| *sample size = 20* | |  |  |  |  |
| --- | --- | --- | --- | --- | --- |
|  | median | mean | sd | t | p.value |
| cor_MRM_region | 0.891765 | 0.888951 | 0.01398 | 0 | 1 |
| cor_MRM_stacked | 0.91119 | 0.909632 | 0.00764 | -5.80538 | 2.58E-06 |
| cor_average | 0.870161 | 0.871569 | 0.011003 | 4.369488 | 0.000101 |
| cor_KNN | 0.85132 | 0.854925 | 0.008875 | 9.189583 | 1.63E-10 |
| cor_RF | 0.877743 | 0.87981 | 0.009639 | 2.407393 | 0.021688 |
| cor_Melissa | 0.490008 | 0.485884 | 0.044019 | 39.02908 | 2.23E-22 |
| *sample size = 50* | |  |  |  |  |
|  | median | mean | sd | t | p.value |
| cor_MRM_region | 0.912275 | 0.911665 | 0.00723 | 0 | 1 |
| cor_MRM_stacked | 0.921428 | 0.920546 | 0.006214 | -6.58725 | 2.41E-09 |
| cor_average | 0.868006 | 0.866736 | 0.009334 | 26.90788 | 1.88E-45 |
| cor_KNN | 0.867917 | 0.867586 | 0.007831 | 29.24325 | 5.08E-50 |
| cor_RF | 0.875463 | 0.874216 | 0.008923 | 23.05832 | 1.84E-40 |
| cor_Melissa | 0.467109 | 0.470561 | 0.054772 | 56.45716 | 1.91E-47 |
| *sample size = 100* | |  |  |  |  |
|  | median | mean | sd | t | p.value |
| cor_MRM_region | 0.921665 | 0.92217 | 0.006637 | 0 | 1 |
| cor_MRM_stacked | 0.929392 | 0.929942 | 0.005499 | -9.01817 | 2.00E-16 |
| cor_average | 0.879266 | 0.877956 | 0.009619 | 37.834 | 2.05E-86 |
| cor_KNN | 0.883155 | 0.883171 | 0.007921 | 37.73829 | 8.67E-91 |
| cor_RF | 0.885575 | 0.885108 | 0.009102 | 32.90181 | 2.59E-78 |
| cor_Melissa | 0.456045 | 0.455622 | 0.040036 | 114.9641 | 8.65E-112 |
| *sample size = 200* | |  |  |  |  |
|  | median | mean | sd | t | p.value |
| cor_MRM_region | 0.924857 | 0.924366 | 0.005858 | 0 | 1 |
| cor_MRM_stacked | 0.930767 | 0.930969 | 0.005299 | -11.8206 | 8.36E-28 |
| cor_average | 0.875904 | 0.875454 | 0.009922 | 60.03351 | 3.68E-177 |
| cor_KNN | 0.884905 | 0.884053 | 0.008241 | 56.38532 | 1.66E-180 |
| cor_RF | 0.881894 | 0.882171 | 0.008672 | 57.01871 | 5.07E-179 |
| cor_Melissa | 0.447074 | 0.446666 | 0.035204 | 189.2977 | 1.25E-236 |
| *t: t-test statistic compared with MRM_region in each setting* | | | | | |

## Table S3b. Test of difference in RMSE under different sample size

| *sample size = 20* | |  |  |  |  |
| --- | --- | --- | --- | --- | --- |
|  | median | mean | sd | t | p.value |
| rmse_MRM_region | 0.124794 | 0.125153 | 0.007248 | 0 | 1 |
| rmse_MRM_stacked | 0.113505 | 0.113861 | 0.004438 | 5.941674 | 1.37E-06 |
| rmse_average | 0.135517 | 0.135305 | 0.004434 | -5.34336 | 7.68E-06 |
| rmse_KNN | 0.142448 | 0.142602 | 0.003154 | -9.8716 | 2.84E-10 |
| rmse_RF | 0.13422 | 0.13555 | 0.004124 | -5.57546 | 4.52E-06 |
| rmse_Melissa | 0.252406 | 0.251292 | 0.008709 | -49.785 | 2.34E-35 |
| *sample size = 50* | |  |  |  |  |
|  | median | mean | sd | t | p.value |
| rmse_MRM_region | 0.112936 | 0.112887 | 0.003942 | 0 | 1 |
| rmse_MRM_stacked | 0.107589 | 0.107408 | 0.003377 | 7.464 | 3.85E-11 |
| rmse_average | 0.1375 | 0.138305 | 0.004086 | -31.6588 | 3.18E-53 |
| rmse_KNN | 0.137243 | 0.137211 | 0.003565 | -32.3639 | 8.60E-54 |
| rmse_RF | 0.137407 | 0.138014 | 0.003952 | -31.8316 | 1.78E-53 |
| rmse_Melissa | 0.252691 | 0.252968 | 0.011742 | -79.9684 | 1.37E-62 |
| *sample size = 100* | |  |  |  |  |
|  | median | mean | sd | t | p.value |
| rmse_MRM_region | 0.107297 | 0.107051 | 0.004177 | 0 | 1 |
| rmse_MRM_stacked | 0.102064 | 0.101827 | 0.003571 | 9.505385 | 8.02E-18 |
| rmse_average | 0.133449 | 0.133512 | 0.004885 | -41.1662 | 1.29E-97 |
| rmse_KNN | 0.130181 | 0.130253 | 0.003666 | -41.7473 | 4.29E-99 |
| rmse_RF | 0.133857 | 0.133782 | 0.004671 | -42.6569 | 5.21E-101 |
| rmse_Melissa | 0.259102 | 0.257472 | 0.010385 | -134.378 | 1.51E-141 |
| *sample size = 200* | |  |  |  |  |
|  | median | mean | sd | t | p.value |
| rmse_MRM_region | 0.104596 | 0.104734 | 0.003476 | 0 | 1 |
| rmse_MRM_stacked | 0.100188 | 0.100268 | 0.003288 | 13.19927 | 3.13E-33 |
| rmse_average | 0.133833 | 0.133802 | 0.004712 | -70.2018 | 1.81E-214 |
| rmse_KNN | 0.128441 | 0.128771 | 0.00391 | -64.9756 | 3.49E-212 |
| rmse_RF | 0.134149 | 0.134207 | 0.004037 | -78.2402 | 2.78E-240 |
| rmse_Melissa | 0.255689 | 0.255935 | 0.008201 | -240.058 | 4.037E-315 |
| *t: t-test statistic compared with MRM_region in each setting* | | | | | |

## Table S3c. Test of difference in AUC under different sample size

| *sample size = 20* | |  |  |  |  |
| --- | --- | --- | --- | --- | --- |
|  | median | mean | sd | t | p.value |
| auc_MRM_region | 0.955903 | 0.955354 | 0.007441 | 0 | 1 |
| auc_MRM_stacked | 0.967312 | 0.966733 | 0.004605 | -5.81561 | 1.92E-06 |
| auc_average | 0.949034 | 0.949739 | 0.006758 | 2.497999 | 0.016978 |
| auc_KNN | 0.945523 | 0.944272 | 0.007734 | 4.617511 | 4.36E-05 |
| auc_RF | 0.954378 | 0.954987 | 0.005629 | 0.175465 | 0.861715 |
| auc_Melissa | 0.781077 | 0.779085 | 0.026798 | 28.34425 | 9.24E-19 |
| *sample size = 50* | |  |  |  |  |
|  | median | mean | sd | t | p.value |
| auc_MRM_region | 0.96562 | 0.965252 | 0.005183 | 0 | 1 |
| auc_MRM_stacked | 0.970655 | 0.96996 | 0.004787 | -4.71839 | 7.95E-06 |
| auc_average | 0.947283 | 0.94681 | 0.00731 | 14.55225 | 3.49E-25 |
| auc_KNN | 0.950697 | 0.949175 | 0.006839 | 13.24835 | 5.56E-23 |
| auc_RF | 0.951745 | 0.951914 | 0.006705 | 11.12862 | 9.78E-19 |
| auc_Melissa | 0.769407 | 0.771321 | 0.032227 | 42.01094 | 1.53E-41 |
| *sample size = 100* | |  |  |  |  |
|  | median | mean | sd | t | p.value |
| auc_MRM_region | 0.97129 | 0.971273 | 0.00556 | 0 | 1 |
| auc_MRM_stacked | 0.975208 | 0.975287 | 0.00481 | -5.46029 | 1.44E-07 |
| auc_average | 0.952349 | 0.95256 | 0.007514 | 20.01826 | 6.59E-48 |
| auc_KNN | 0.956548 | 0.956154 | 0.006244 | 18.08346 | 1.35E-43 |
| auc_RF | 0.957716 | 0.957537 | 0.006864 | 15.55101 | 1.05E-35 |
| auc_Melissa | 0.762136 | 0.762267 | 0.023964 | 84.96093 | 7.00E-102 |
| *sample size = 200* | |  |  |  |  |
|  | median | mean | sd | t | p.value |
| auc_MRM_region | 0.971737 | 0.971034 | 0.004904 | 0 | 1 |
| auc_MRM_stacked | 0.975337 | 0.974695 | 0.004384 | -7.87053 | 3.46E-14 |
| auc_average | 0.950768 | 0.950833 | 0.00719 | 32.82538 | 4.81E-109 |
| auc_KNN | 0.95674 | 0.956599 | 0.006239 | 25.72431 | 5.55E-85 |
| auc_RF | 0.955763 | 0.955635 | 0.006563 | 26.58301 | 1.09E-87 |
| auc_Melissa | 0.756574 | 0.755988 | 0.021782 | 136.2102 | 9.40E-214 |
| *t: t-test statistic compared with MRM_region in each setting* | | | | | |

## Table S4a. Test of difference in correlation under different missing rate

| *missing rate = 0.2* | |  |  |  |  |
| --- | --- | --- | --- | --- | --- |
|  | median | mean | sd | t | p.value |
| cor_MRM_region | 0.891765 | 0.888951 | 0.01398 | 0 | 1 |
| cor_MRM_stacked | 0.91119 | 0.909632 | 0.00764 | -5.80538 | 2.58E-06 |
| cor_average | 0.870161 | 0.871569 | 0.011003 | 4.369488 | 0.000101 |
| cor_KNN | 0.85132 | 0.854925 | 0.008875 | 9.189583 | 1.63E-10 |
| cor_RF | 0.877743 | 0.87981 | 0.009639 | 2.407393 | 0.021688 |
| cor_Melissa | 0.490008 | 0.485884 | 0.044019 | 39.02908 | 2.23E-22 |
| *missing rate = 0.4* | |  |  |  |  |
|  | median | mean | sd | t | p.value |
| cor_MRM_region | 0.860341 | 0.861587 | 0.014627 | 0 | 1 |
| cor_MRM_stacked | 0.887479 | 0.889456 | 0.00778 | -7.52281 | 2.75E-08 |
| cor_average | 0.843247 | 0.843351 | 0.007412 | 4.9736 | 2.94E-05 |
| cor_KNN | 0.816378 | 0.814485 | 0.010627 | 11.6508 | 1.53E-13 |
| cor_RF | 0.867331 | 0.869351 | 0.007516 | -2.11141 | 0.043669 |
| cor_Melissa | 0.473668 | 0.475727 | 0.047636 | 34.62912 | 4.73E-21 |
| *missing rate = 0.6* | |  |  |  |  |
|  | median | mean | sd | t | p.value |
| cor_MRM_region | 0.83661 | 0.830524 | 0.01638 | 0 | 1 |
| cor_MRM_stacked | 0.864632 | 0.86164 | 0.013482 | -6.55956 | 1.15E-07 |
| cor_average | 0.777325 | 0.77679 | 0.014236 | 11.07326 | 2.40E-13 |
| cor_KNN | 0.678478 | 0.677934 | 0.013101 | 32.53491 | 2.00E-28 |
| cor_RF | 0.846207 | 0.844662 | 0.009509 | -3.33849 | 0.002231 |
| cor_Melissa | 0.47094 | 0.473989 | 0.031182 | 45.26872 | 2.84E-28 |
| *missing rate = 0.8* | |  |  |  |  |
|  | median | mean | sd | t | p.value |
| cor_MRM_region | 0.783688 | 0.781771 | 0.020282 | 0 | 1 |
| cor_MRM_stacked | 0.803692 | 0.799552 | 0.016558 | -3.03713 | 0.00439 |
| cor_average | 0.571253 | 0.565948 | 0.024622 | 30.25646 | 1.58E-27 |
| cor_KNN | 0.347198 | 0.343274 | 0.017967 | 72.37292 | 7.47E-42 |
| cor_RF | 0.764862 | 0.763213 | 0.015512 | 3.250235 | 0.002524 |
| cor_Melissa | 0.441879 | 0.43887 | 0.033439 | 39.21015 | 3.48E-28 |
| *t: t-test statistic compared with MRM_region in each setting* | | | | | |

## Table S4b. Test of difference in RMSE under different missing rate

| *missing rate = 0.2* | |  |  |  |  |
| --- | --- | --- | --- | --- | --- |
|  | median | mean | sd | t | p.value |
| rmse_MRM_region | 0.124794 | 0.125153 | 0.007248 | 0 | 1 |
| rmse_MRM_stacked | 0.113505 | 0.113861 | 0.004438 | 5.941674 | 1.37E-06 |
| rmse_average | 0.135517 | 0.135305 | 0.004434 | -5.34336 | 7.68E-06 |
| rmse_KNN | 0.142448 | 0.142602 | 0.003154 | -9.8716 | 2.84E-10 |
| rmse_RF | 0.13422 | 0.13555 | 0.004124 | -5.57546 | 4.52E-06 |
| rmse_Melissa | 0.252406 | 0.251292 | 0.008709 | -49.785 | 2.34E-35 |
| *missing rate = 0.4* | |  |  |  |  |
|  | median | mean | sd | t | p.value |
| rmse_MRM_region | 0.140096 | 0.13878 | 0.007029 | 0 | 1 |
| rmse_MRM_stacked | 0.1259 | 0.125253 | 0.004358 | 7.314149 | 2.72E-08 |
| rmse_average | 0.148945 | 0.148833 | 0.003194 | -5.82278 | 3.61E-06 |
| rmse_KNN | 0.158644 | 0.159086 | 0.003838 | -11.3383 | 2.96E-12 |
| rmse_RF | 0.13755 | 0.13764 | 0.003344 | 0.654951 | 0.518004 |
| rmse_Melissa | 0.253632 | 0.252671 | 0.009466 | -43.1972 | 5.30E-32 |
| *missing rate = 0.6* | |  |  |  |  |
|  | median | mean | sd | t | p.value |
| rmse_MRM_region | 0.150011 | 0.152236 | 0.00623 | 0 | 1 |
| rmse_MRM_stacked | 0.137922 | 0.138971 | 0.005617 | 7.07201 | 2.08E-08 |
| rmse_average | 0.174969 | 0.175763 | 0.004822 | -13.3547 | 1.84E-15 |
| rmse_KNN | 0.20161 | 0.201501 | 0.003305 | -31.2402 | 7.91E-24 |
| rmse_RF | 0.146877 | 0.147637 | 0.004067 | 2.76405 | 0.009303 |
| rmse_Melissa | 0.251956 | 0.250985 | 0.007453 | -45.4629 | 5.76E-34 |
| *missing rate = 0.8* | |  |  |  |  |
|  | median | mean | sd | t | p.value |
| rmse_MRM_region | 0.171523 | 0.170866 | 0.006898 | 0 | 1 |
| rmse_MRM_stacked | 0.164674 | 0.16472 | 0.005961 | 3.014854 | 0.004611 |
| rmse_average | 0.237158 | 0.238127 | 0.0057 | -33.6141 | 3.62E-29 |
| rmse_KNN | 0.283514 | 0.283485 | 0.004261 | -62.1156 | 1.19E-34 |
| rmse_RF | 0.178039 | 0.178005 | 0.00468 | -3.83007 | 0.000536 |
| rmse_Melissa | 0.253644 | 0.254891 | 0.00846 | -34.4251 | 1.94E-29 |
| *t: t-test statistic compared with MRM_region in each setting* | | | | | |

## Table S4c. Test of difference in AUC under different missing rate

| *missing rate = 0.2* | |  |  |  |  |
| --- | --- | --- | --- | --- | --- |
|  | median | mean | sd | t | p.value |
| auc_MRM_region | 0.955903 | 0.955354 | 0.007441 | 0 | 1 |
| auc_MRM_stacked | 0.967312 | 0.966733 | 0.004605 | -5.81561 | 1.92E-06 |
| auc_average | 0.949034 | 0.949739 | 0.006758 | 2.497999 | 0.016978 |
| auc_KNN | 0.945523 | 0.944272 | 0.007734 | 4.617511 | 4.36E-05 |
| auc_RF | 0.954378 | 0.954987 | 0.005629 | 0.175465 | 0.861715 |
| auc_Melissa | 0.781077 | 0.779085 | 0.026798 | 28.34425 | 9.24E-19 |
| *missing rate = 0.4* | |  |  |  |  |
|  | median | mean | sd | t | p.value |
| auc_MRM_region | 0.942372 | 0.944164 | 0.007824 | 0 | 1 |
| auc_MRM_stacked | 0.958157 | 0.958623 | 0.005375 | -6.81206 | 8.15E-08 |
| auc_average | 0.934965 | 0.935707 | 0.003824 | 4.34293 | 0.000171 |
| auc_KNN | 0.924934 | 0.924706 | 0.007247 | 8.15938 | 7.36E-10 |
| auc_RF | 0.948888 | 0.949363 | 0.004305 | -2.60344 | 0.014295 |
| auc_Melissa | 0.770395 | 0.773713 | 0.028174 | 26.06959 | 5.49E-18 |
| *missing rate = 0.6* | |  |  |  |  |
|  | median | mean | sd | t | p.value |
| auc_MRM_region | 0.929815 | 0.927344 | 0.009938 | 0 | 1 |
| auc_MRM_stacked | 0.945622 | 0.944837 | 0.007764 | -6.20341 | 3.76E-07 |
| auc_average | 0.903547 | 0.902026 | 0.010225 | 7.941168 | 1.37E-09 |
| auc_KNN | 0.857507 | 0.858306 | 0.008739 | 23.33053 | 6.97E-24 |
| auc_RF | 0.938916 | 0.938216 | 0.006917 | -4.01559 | 0.000311 |
| auc_Melissa | 0.770631 | 0.772829 | 0.018602 | 32.76474 | 1.76E-24 |
| *missing rate = 0.8* | |  |  |  |  |
|  | median | mean | sd | t | p.value |
| auc_MRM_region | 0.905649 | 0.90553 | 0.011321 | 0 | 1 |
| auc_MRM_stacked | 0.917828 | 0.917275 | 0.009093 | -3.61712 | 0.000899 |
| auc_average | 0.799125 | 0.796035 | 0.01662 | 24.35058 | 6.93E-23 |
| auc_KNN | 0.685027 | 0.685486 | 0.009395 | 66.89145 | 5.39E-40 |
| auc_RF | 0.902464 | 0.901066 | 0.008829 | 1.390712 | 0.172881 |
| auc_Melissa | 0.755764 | 0.752604 | 0.019935 | 29.83174 | 6.50E-24 |
| *t: t-test statistic compared with MRM_region in each setting* | | | | | |

## Table S5a. Test of difference in correlation under different missing rate in pWGBSSimla simulated data (N=20)

| *missing rate = 0.2* |  |  |  |  |  |
| --- | --- | --- | --- | --- | --- |
|  | median | mean | sd | statistic | p.value |
| cor_MRM_region | 0.462192 | 0.462161 | 0.05912 | 0 | 1 |
| cor_MRM_stacked | 0.449864 | 0.438307 | 0.066711 | 6.844031 | 9.10E-12 |
| cor_average | 0.300671 | 0.3017 | 0.066115 | 10.09655 | 1.23E-23 |
| cor_KNN | 0.350657 | 0.359712 | 0.068293 | 12.22036 | 1.22E-33 |
| cor_RF | 0.298987 | 0.28972 | 0.071022 | 13.44039 | 3.66E-40 |
| cor_Melissa | 0.380325 | 0.387071 | 0.058487 | 7.602458 | 3.74E-14 |
| *missing rate = 0.4* |  |  |  |  |  |
|  | median | mean | sd | statistic | p.value |
| cor_MRM_region | 0.464999 | 0.471083 | 0.057388 | 0 | 1 |
| cor_MRM_stacked | 0.454914 | 0.456921 | 0.056987 | 5.236273 | 1.69E-07 |
| cor_average | 0.296815 | 0.301411 | 0.054127 | 13.31419 | 6.04E-40 |
| cor_KNN | 0.348827 | 0.360204 | 0.057584 | 18.50273 | 1.30E-74 |
| cor_RF | 0.282808 | 0.286475 | 0.04999 | 19.3095 | 6.37E-81 |
| cor_Melissa | 0.386459 | 0.372431 | 0.050619 | 13.15275 | 4.90E-39 |
| *missing rate = 0.6* |  |  |  |  |  |
|  | median | mean | sd | statistic | p.value |
| cor_MRM_region | 0.462715 | 0.465057 | 0.025864 | NaN | NaN |
| cor_MRM_stacked | 0.440955 | 0.435098 | 0.037373 | 9.957232 | 2.99E-23 |
| cor_average | 0.292572 | 0.280481 | 0.060986 | 21.68351 | 5.77E-102 |
| cor_KNN | 0.306167 | 0.300113 | 0.048673 | 24.15162 | 2.28E-125 |
| cor_RF | 0.276222 | 0.267896 | 0.062143 | 24.16055 | 1.86E-125 |
| cor_Melissa | 0.396668 | 0.407786 | 0.036033 | 10.04911 | 1.19E-23 |
| *missing rate = 0.8* |  |  |  |  |  |
|  | median | mean | sd | statistic | p.value |
| cor_MRM_region | 0.438758 | 0.43396 | 0.044909 | 0 | 1 |
| cor_MRM_stacked | 0.392586 | 0.395691 | 0.046299 | 11.82307 | 4.26E-32 |
| cor_average | 0.278757 | 0.274248 | 0.058361 | 23.23143 | 4.12E-117 |
| cor_KNN | 0.247696 | 0.258432 | 0.043781 | 22.05692 | 5.85E-106 |
| cor_RF | 0.266441 | 0.269551 | 0.064248 | 23.01439 | 5.20E-115 |
| cor_Melissa | 0.390345 | 0.383253 | 0.03811 | 8.045636 | 9.29E-16 |

## Table S5b. Test of difference in RMSE under different missing rate in pWGBSSimla simulated data (N=20)

| *missing rate = 0.2* |  |  |  |  |  |
| --- | --- | --- | --- | --- | --- |
|  | median | mean | sd | statistic | p.value |
| rmse_MRM_region | 0.132453 | 0.130488 | 0.009825 | NA | NA |
| rmse_MRM_stacked | 0.133834 | 0.132951 | 0.00977 | -6.76388 | 1.58E-11 |
| rmse_average | 0.158014 | 0.159223 | 0.008413 | -10.9725 | 1.49E-27 |
| rmse_KNN | 0.142637 | 0.140785 | 0.00918 | -17.4001 | 5.09E-65 |
| rmse_RF | 0.142442 | 0.140839 | 0.009398 | -9.97262 | 4.17E-23 |
| rmse_Melissa | 0.182615 | 0.178665 | 0.012124 | -23.7554 | 1.63E-115 |
| *missing rate = 0.4* |  |  |  |  |  |
|  | median | mean | sd | statistic | p.value |
| rmse_MRM_region | 0.131539 | 0.131329 | 0.008335 | NA | NA |
| rmse_MRM_stacked | 0.131534 | 0.132756 | 0.007796 | -4.86916 | 1.15E-06 |
| rmse_average | 0.160752 | 0.161204 | 0.00754 | -13.6054 | 1.30E-41 |
| rmse_KNN | 0.143334 | 0.142159 | 0.00805 | -24.2499 | 1.21E-124 |
| rmse_RF | 0.143979 | 0.142734 | 0.006244 | -14.8545 | 3.84E-49 |
| rmse_Melissa | 0.18053 | 0.178145 | 0.01099 | -32.1648 | 2.30E-211 |
| *missing rate = 0.6* |  |  |  |  |  |
|  | median | mean | sd | statistic | p.value |
| rmse_MRM_region | 0.131305 | 0.132764 | 0.005322 | NA | NA |
| rmse_MRM_stacked | 0.135672 | 0.135704 | 0.005651 | -9.96667 | 2.73E-23 |
| rmse_average | 0.164989 | 0.165442 | 0.007979 | -24.8192 | 4.38E-132 |
| rmse_KNN | 0.152808 | 0.152946 | 0.005111 | -30.4542 | 5.47E-195 |
| rmse_RF | 0.145193 | 0.144613 | 0.004814 | -18.3416 | 6.02E-74 |
| rmse_Melissa | 0.178169 | 0.179023 | 0.0058 | -39.693 | 2.25886813278618e-320 |
| *missing rate = 0.8* |  |  |  |  |  |
|  | median | mean | sd | statistic | p.value |
| rmse_MRM_region | 0.135349 | 0.137115 | 0.005574 | NA | NA |
| rmse_MRM_stacked | 0.141422 | 0.141377 | 0.005519 | -13.3176 | 3.27E-40 |
| rmse_average | 0.166543 | 0.167452 | 0.007932 | -27.9145 | 8.56E-167 |
| rmse_KNN | 0.166005 | 0.166582 | 0.007437 | -31.4657 | 9.37E-210 |
| rmse_RF | 0.144898 | 0.146464 | 0.004995 | -15.808 | 8.71E-56 |
| rmse_Melissa | 0.17927 | 0.179527 | 0.004999 | -42.0942 | 0 |

## Table S5c. Test of difference in AUC under different missing rate in pWGBSSimla simulated data (N=20)

| *missing rate = 0.2* |  |  |  |  |  |
| --- | --- | --- | --- | --- | --- |
|  | median | mean | sd | statistic | p.value |
| auc_MRM_region | 0.709414 | 0.684109 | 0.068618 | 0 | 1 |
| auc_MRM_stacked | 0.695744 | 0.670656 | 0.074117 | 2.116061 | 0.03434 |
| auc_average | 0.589152 | 0.59423 | 0.09548 | 2.579541 | 0.009893 |
| auc_KNN | 0.657793 | 0.640388 | 0.08133 | 4.059102 | 4.93E-05 |
| auc_RF | 0.615955 | 0.602659 | 0.096951 | 3.90968 | 0.862675 |
| auc_Melissa | 0.697118 | 0.690268 | 0.081444 | -0.17297 | 0.862675 |
| *missing rate = 0.4* |  |  |  |  |  |
|  | median | mean | sd | statistic | p.value |
| auc_MRM_region | 0.690535 | 0.678548 | 0.106078 | 0 | 1 |
| auc_MRM_stacked | 0.694502 | 0.677887 | 0.104782 | 0.575564 | 0.56491 |
| auc_average | 0.627661 | 0.620499 | 0.09129 | 1.705427 | 0.088115 |
| auc_KNN | 0.660903 | 0.656137 | 0.076557 | 4.19109 | 2.78E-05 |
| auc_RF | 0.602091 | 0.61049 | 0.09003 | 4.218248 | 2.46E-05 |
| auc_Melissa | 0.670956 | 0.675589 | 0.076926 | 0.064634 | 0.948466 |
| *missing rate = 0.6* |  |  |  |  |  |
|  | median | mean | sd | statistic | p.value |
| auc_MRM_region | 0.666784 | 0.669112 | 0.071181 | 0 | 1 |
| auc_MRM_stacked | 0.640089 | 0.659296 | 0.06634 | 1.644628 | 0.100047 |
| auc_average | 0.630284 | 0.62563 | 0.068453 | 3.772089 | 0.000162 |
| auc_KNN | 0.613183 | 0.621964 | 0.088321 | 3.899961 | 9.62E-05 |
| auc_RF | 0.622198 | 0.608697 | 0.073631 | 4.150684 | 3.31E-05 |
| auc_Melissa | 0.667836 | 0.657629 | 0.083736 | 1.048803 | 0.294269 |
| *missing rate = 0.8* |  |  |  |  |  |
|  | median | mean | sd | statistic | p.value |
| auc_MRM_region | 0.687537 | 0.680039 | 0.073277 | 0 | 1 |
| auc_MRM_stacked | 0.669227 | 0.666454 | 0.070793 | 3.030364 | 0.002443 |
| auc_average | 0.634325 | 0.621736 | 0.068389 | 5.367843 | 7.97E-08 |
| auc_KNN | 0.621117 | 0.617657 | 0.058948 | 5.575492 | 2.47E-08 |
| auc_RF | 0.611678 | 0.606337 | 0.068026 | 6.076916 | 1.23E-09 |
| auc_Melissa | 0.675177 | 0.678654 | 0.071819 | 0.421011 | 0.673747 |

## Table S5d. Test of difference in correlation under different missing rate in pWGBSSimla simulated data (N=100)

| *missing rate = 0.2* |  |  |  |  |  |
| --- | --- | --- | --- | --- | --- |
|  | median | mean | sd | statistic | p.value |
| cor_MRM_region | 0.485787 | 0.480457 | 0.067958 | 0 | 1 |
| cor_MRM_stacked | 0.472127 | 0.461805 | 0.067558 | 9.832004 | 9.43E-23 |
| cor_average | 0.308144 | 0.303551 | 0.082139 | 19.01958 | 7.92E-80 |
| cor_KNN | 0.413559 | 0.403036 | 0.066629 | 30.21268 | 2.28E-195 |
| cor_RF | 0.318534 | 0.305934 | 0.078808 | 29.85935 | 5.50E-191 |
| cor_Melissa | 0.447666 | 0.434639 | 0.073964 | 13.02314 | 1.38E-38 |
| *missing rate = 0.4* |  |  |  |  |  |
|  | median | mean | sd | statistic | p.value |
| cor_MRM_region | 0.485816 | 0.488382 | 0.047435 | 0 | 1 |
| cor_MRM_stacked | 0.457024 | 0.462333 | 0.048405 | 17.56845 | 8.66E-69 |
| cor_average | 0.303795 | 0.30096 | 0.05669 | 33.41974 | 5.70E-241 |
| cor_KNN | 0.384345 | 0.381718 | 0.047723 | 45.23434 | 0 |
| cor_RF | 0.292324 | 0.290463 | 0.055626 | 46.24892 | 0 |
| cor_Melissa | 0.436485 | 0.439281 | 0.046589 | 19.35709 | 4.98E-83 |
| *missing rate = 0.6* |  |  |  |  |  |
|  | median | mean | sd | statistic | p.value |
| cor_MRM_region | 0.486257 | 0.483274 | 0.035078 | 0 | 1 |
| cor_MRM_stacked | 0.450906 | 0.451687 | 0.040402 | 23.06425 | 4.22E-117 |
| cor_average | 0.293551 | 0.2922 | 0.051623 | 47.92933 | 0 |
| cor_KNN | 0.32963 | 0.333679 | 0.047347 | 55.08283 | 0 |
| cor_RF | 0.276733 | 0.278084 | 0.059459 | 56.62169 | 0 |
| cor_Melissa | 0.436918 | 0.432492 | 0.039223 | 22.03555 | 4.16E-107 |
| *missing rate = 0.8* |  |  |  |  |  |
|  | median | mean | sd | statistic | p.value |
| cor_MRM_region | 0.47145 | 0.473448 | 0.030364 | 0 | 1 |
| cor_MRM_stacked | 0.426412 | 0.425282 | 0.041211 | 33.82764 | 9.12E-249 |
| cor_average | 0.273689 | 0.274126 | 0.046567 | 65.96201 | 0 |
| cor_KNN | 0.257286 | 0.260188 | 0.046493 | 63.36197 | 0 |
| cor_RF | 0.250864 | 0.255156 | 0.063863 | 65.86542 | 0 |
| cor_Melissa | 0.421972 | 0.420708 | 0.035211 | 23.73609 | 4.90E-124 |

## Table S5e. Test of difference in RMSE under different missing rate in pWGBSSimla simulated data (N=100)

| *missing rate = 0.2* |  |  |  |  |  |
| --- | --- | --- | --- | --- | --- |
|  | median | mean | sd | statistic | p.value |
| rmse_MRM_region | 0.133691 | 0.134103 | 0.011979 | NA | NA |
| rmse_MRM_stacked | 0.13523 | 0.135778 | 0.011836 | -9.62682 | 7.01E-22 |
| rmse_average | 0.161721 | 0.16336 | 0.012056 | -20.2083 | 9.35E-90 |
| rmse_KNN | 0.140894 | 0.142127 | 0.011568 | -38.127 | 3.20E-305 |
| rmse_RF | 0.145915 | 0.145544 | 0.011593 | -23.7833 | 5.15E-123 |
| rmse_Melissa | 0.185128 | 0.184591 | 0.015126 | -55.71 | 0 |
| *missing rate = 0.4* |  |  |  |  |  |
|  | median | mean | sd | statistic | p.value |
| rmse_MRM_region | 0.132334 | 0.133305 | 0.006748 | NA | NA |
| rmse_MRM_stacked | 0.135102 | 0.135653 | 0.006735 | -16.9967 | 1.61E-64 |
| rmse_average | 0.164872 | 0.16481 | 0.008169 | -35.2309 | 4.35E-267 |
| rmse_KNN | 0.144502 | 0.144662 | 0.006977 | -55.5089 | 0 |
| rmse_RF | 0.145037 | 0.146226 | 0.006428 | -37.6452 | 7.44E-304 |
| rmse_Melissa | 0.183168 | 0.183794 | 0.008672 | -77.5341 | 0 |
| *missing rate = 0.6* |  |  |  |  |  |
|  | median | mean | sd | statistic | p.value |
| rmse_MRM_region | 0.133507 | 0.133744 | 0.005738 | NA | NA |
| rmse_MRM_stacked | 0.137128 | 0.136721 | 0.00585 | -22.7645 | 3.83E-114 |
| rmse_average | 0.165702 | 0.166262 | 0.007895 | -51.3704 | 0 |
| rmse_KNN | 0.151322 | 0.151811 | 0.006082 | -66.8748 | 0 |
| rmse_RF | 0.146726 | 0.146941 | 0.006036 | -45.4222 | 0 |
| rmse_Melissa | 0.183797 | 0.183161 | 0.007511 | -93.7881 | 0 |
| *missing rate = 0.8* |  |  |  |  |  |
|  | median | mean | sd | statistic | p.value |
| rmse_MRM_region | 0.134318 | 0.134885 | 0.004789 | NA | NA |
| rmse_MRM_stacked | 0.139448 | 0.139588 | 0.005375 | -34.3253 | 5.16E-256 |
| rmse_average | 0.168643 | 0.168463 | 0.007345 | -72.8814 | 0 |
| rmse_KNN | 0.166819 | 0.167078 | 0.006795 | -77.161 | 0 |
| rmse_RF | 0.14825 | 0.14832 | 0.005317 | -51.7929 | 0 |
| rmse_Melissa | 0.18175 | 0.182649 | 0.005707 | -105.879 | 0 |

## Table S5f. Test of difference in AUC under different missing rate in pWGBSSimla simulated data (N=100)

| *missing rate = 0.2* |  |  |  |  |  |
| --- | --- | --- | --- | --- | --- |
|  | median | mean | sd | statistic | p.value |
| auc_MRM_region | 0.71144 | 0.700337 | 0.10933 | 0 | 1 |
| auc_MRM_stacked | 0.71378 | 0.694007 | 0.108377 | 2.580663 | 0.009861 |
| auc_average | 0.629584 | 0.61924 | 0.113535 | 2.951557 | 0.003162 |
| auc_KNN | 0.686702 | 0.685815 | 0.106846 | 8.359644 | 6.29E-17 |
| auc_RF | 0.62004 | 0.618993 | 0.109856 | 7.58416 | 3.35E-14 |
| auc_Melissa | 0.708502 | 0.697931 | 0.111328 | 0.107677 | 0.914252 |
| *missing rate = 0.4* |  |  |  |  |  |
|  | median | mean | sd | statistic | p.value |
| auc_MRM_region | 0.711755 | 0.710865 | 0.086354 | 0 | 1 |
| auc_MRM_stacked | 0.697815 | 0.701694 | 0.081929 | 4.697256 | 2.64E-06 |
| auc_average | 0.645167 | 0.636485 | 0.084489 | 7.615494 | 2.63E-14 |
| auc_KNN | 0.666362 | 0.67413 | 0.083097 | 11.40082 | 4.14E-30 |
| auc_RF | 0.654508 | 0.638865 | 0.081216 | 10.25483 | 1.13E-24 |
| auc_Melissa | 0.720777 | 0.711331 | 0.082316 | 0.111875 | 0.910922 |
| *missing rate = 0.6* |  |  |  |  |  |
|  | median | mean | sd | statistic | p.value |
| auc_MRM_region | 0.721837 | 0.716993 | 0.065428 | 0 | 1 |
| auc_MRM_stacked | 0.697922 | 0.700931 | 0.067021 | 6.948175 | 3.70E-12 |
| auc_average | 0.646916 | 0.645775 | 0.066499 | 13.15958 | 1.50E-39 |
| auc_KNN | 0.649995 | 0.654201 | 0.059189 | 12.98354 | 1.52E-38 |
| auc_RF | 0.627833 | 0.621639 | 0.071329 | 15.11913 | 1.21E-51 |
| auc_Melissa | 0.707442 | 0.706623 | 0.063422 | 3.250699 | 0.001151 |
| *missing rate = 0.8* |  |  |  |  |  |
|  | median | mean | sd | statistic | p.value |
| auc_MRM_region | 0.709526 | 0.710665 | 0.054272 | 0 | 1 |
| auc_MRM_stacked | 0.695999 | 0.696654 | 0.053769 | 6.830153 | 8.48E-12 |
| auc_average | 0.636426 | 0.638295 | 0.059225 | 16.83719 | 1.30E-63 |
| auc_KNN | 0.624697 | 0.624862 | 0.057732 | 14.64389 | 1.47E-48 |
| auc_RF | 0.621552 | 0.624429 | 0.062754 | 15.40694 | 1.47E-53 |
| auc_Melissa | 0.703998 | 0.704103 | 0.053647 | 2.500175 | 0.012413 |

## Table S6a. Test of difference in correlation of MRM regional model under different real data preprocessing conditions

| *missing rate = 0.2* | |  |  |  |
| --- | --- | --- | --- | --- |
|  | mean1 | mean2 | t | p.value |
| condition 0-1 | 0.851973088 | 0.623831407 | 19.88897 | 4.15E-20 |
| condition 0-2 | 0.851973088 | 0.767806896 | 7.33914 | 1.84E-08 |
| condition 1-2 | 0.623831407 | 0.767806896 | -11.1144 | 3.43E-13 |
| *missing rate = 0.4* | |  |  |  |
|  | mean1 | mean2 | t | p.value |
| condition 0-1 | 0.836661155 | 0.612883003 | 9.326633 | 4.59E-10 |
| condition 0-2 | 0.836661155 | 0.767153405 | 3.150582 | 0.004733 |
| condition 1-2 | 0.612883003 | 0.767153405 | -11.6387 | 2.23E-12 |
| *missing rate = 0.6* | |  |  |  |
|  | mean1 | mean2 | t | p.value |
| condition 0-1 | 0.847765808 | 0.591035122 | 28.55753 | 3.59E-20 |
| condition 0-2 | 0.847765808 | 0.757393008 | 10.38758 | 1.69E-10 |
| condition 1-2 | 0.591035122 | 0.757393008 | -14.479 | 1.43E-16 |
| *missing rate = 0.8* | |  |  |  |
|  | mean1 | mean2 | t | p.value |
| condition 0-1 | 0.819511648 | 0.542180918 | 24.71137 | 3.59E-24 |
| condition 0-2 | 0.819511648 | 0.708441704 | 11.75119 | 7.40E-13 |
| condition 1-2 | 0.542180918 | 0.708441704 | -17.714 | 1.14E-17 |

## Table S6b. Test of difference in correlation of MRM stacked model under different real data preprocessing conditions

| *mssing rate = 0.2* | |  | |  | |  | |
| --- | --- | --- | --- | --- | --- | --- | --- |
|  | mean1 | | mean2 | | t | | p.value |
| condition 0-1 | 0.846045 | | 0.604914 | | 17.3608 | | 2.73E-17 |
| condition 0-2 | 0.846045 | | 0.761109 | | 7.033411 | | 4.15E-08 |
| condition 1-2 | 0.604914 | | 0.761109 | | -10.3076 | | 4.44E-12 |
| *mssing rate = 0.4* | |  | |  | |  | |
|  | mean1 | | mean2 | | t | | p.value |
| condition 0-1 | 0.82821 | | 0.583 | | 9.545173 | | 1.14E-10 |
| condition 0-2 | 0.82821 | | 0.755487 | | 3.177271 | | 0.004352 |
| condition 1-2 | 0.583 | | 0.755487 | | -11.0619 | | 1.29E-11 |
| *mssing rate = 0.6* | |  | |  | |  | |
|  | mean1 | | mean2 | | t | | p.value |
| condition 0-1 | 0.83705 | | 0.562906 | | 28.92916 | | 1.97E-21 |
| condition 0-2 | 0.83705 | | 0.741913 | | 10.26516 | | 9.35E-11 |
| condition 1-2 | 0.562906 | | 0.741913 | | -15.0913 | | 3.89E-17 |
| *mssing rate = 0.8* | |  | |  | |  | |
|  | mean1 | | mean2 | | t | | p.value |
| condition 0-1 | 0.803355 | | 0.510585 | | 21.38933 | | 1.63E-20 |
| condition 0-2 | 0.803355 | | 0.684423 | | 11.58374 | | 1.44E-13 |
| condition 1-2 | 0.510585 | | 0.684423 | | -13.2634 | | 6.38E-14 |

## Table S7. List of 42 genes, promoter region of which contain more than 10 DMCs.

| Gene | Number of DMCs |
| --- | --- |
| SLC4A1 | 63 |
| GPS2 | 36 |
| KIAA0195 | 34 |
| RP11-798G7 | 31 |
| KCNJ12 | 30 |
| CYTH1 | 29 |
| TNFRSF13B | 29 |
| PLXDC1 | 28 |
| FAM211A | 26 |
| FBXO39 | 24 |
| SPATA20* | 23 |
| RP11-1260E13 | 21 |
| CASKIN2 | 19 |
| PEMT | 18 |
| SLC38A10 | 18 |
| SPACA3 | 18 |
| PFAS | 17 |
| CARD14 | 16 |
| DVL2 | 16 |
| LINC00483 | 16 |
| RNU6-862P | 16 |
| SPECC1* | 16 |
| TMEM97 | 16 |
| MGAT5B | 15 |
| ATP5G1 | 14 |
| CHD3 | 14 |
| GNGT2 | 14 |
| ITGAE | 14 |
| ACE | 13 |
| MYO1D* | 13 |
| SCN4A | 13 |
| SMG6 | 13 |
| ABCC3 | 12 |
| ATP1B2* | 12 |
| C17orf70 | 12 |
| C1QBP | 12 |
| KRT15 | 12 |
| RP11-744K17 | 12 |
| VPS25 | 12 |
| DNAI2 | 11 |
| HOXB3 | 11 |
| USP36* | 11 |

* Genes that were associated with BMD or bone metabolism

## Table S8. Computation time (min) under different sample size on a 4-core core i7 desk top

| Sample size | Run Time (min) | | | | | | |
| --- | --- | --- | --- | --- | --- | --- | --- |
|  | MRM_region | MRM_subj | MRM_stacking | average | KNN | RF | Melissa |
| 20 | 1.56 | 1.29 | 0.60 | 0.02 | 0.14 | 0.40 | 0.11 |
| 50 | 1.98 | 2.75 | 1.47 | 0.06 | 0.28 | 1.05 | 0.25 |
| 100 | 2.84 | 5.13 | 2.91 | 0.11 | 0.53 | 2.69 | 0.52 |
| 200 | 5.74 | 10.49 | 5.80 | 0.23 | 2.79 | 4.66 | 1.18 |

# Figures


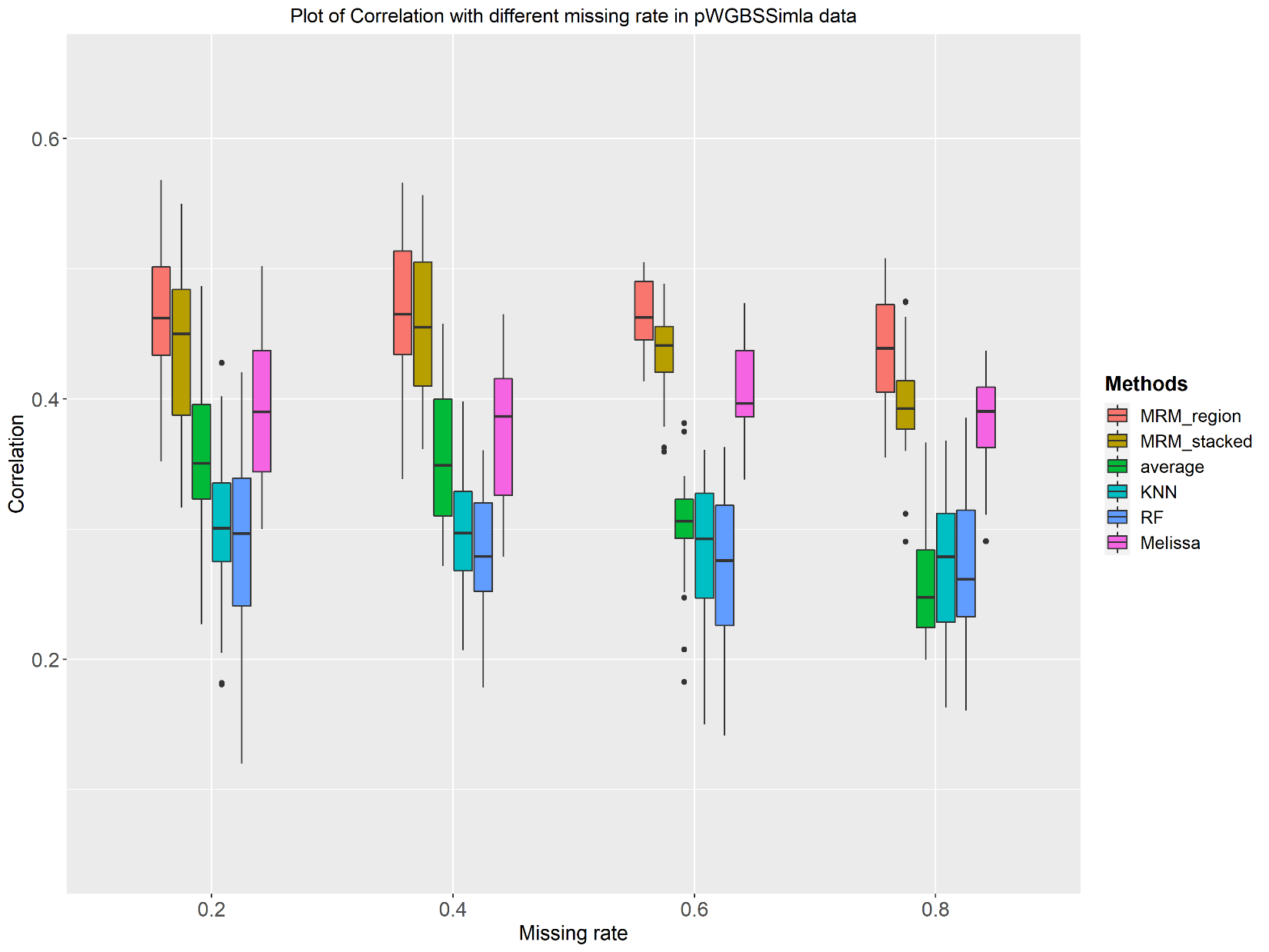


## Figure S1a. Box plot of correlation of six imputation algorithms on pWGBSSimla simulated data under the effect of the missing rate. Each colored boxplot indicates the imputation performance on N=20 subjects.


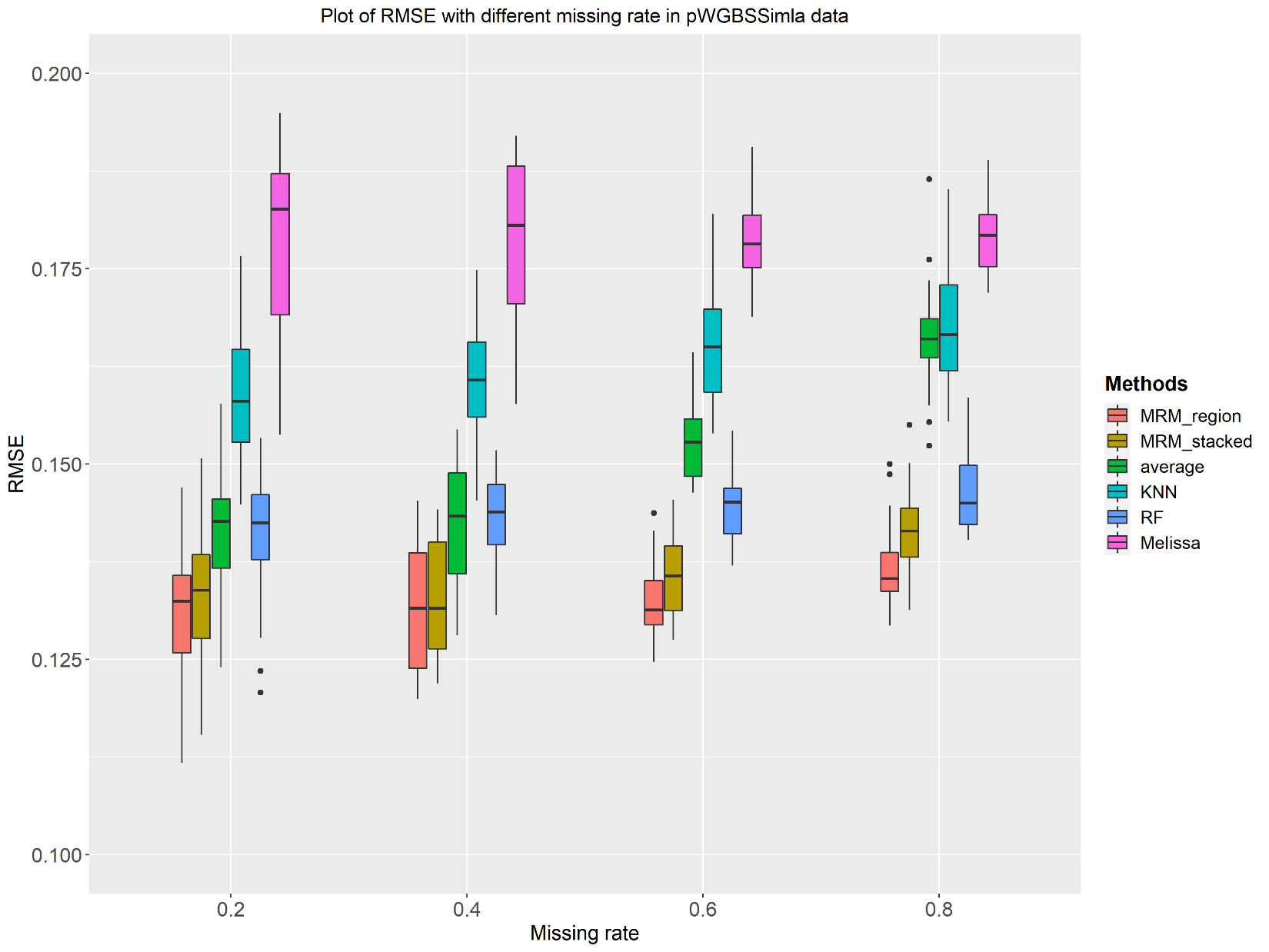


## Figure S1b. Box plot of RMSE of six imputation algorithms on pWGBSSimla simulated data under the effect of the missing rate. Each colored boxplot indicates the imputation performance on N=20 subjects.


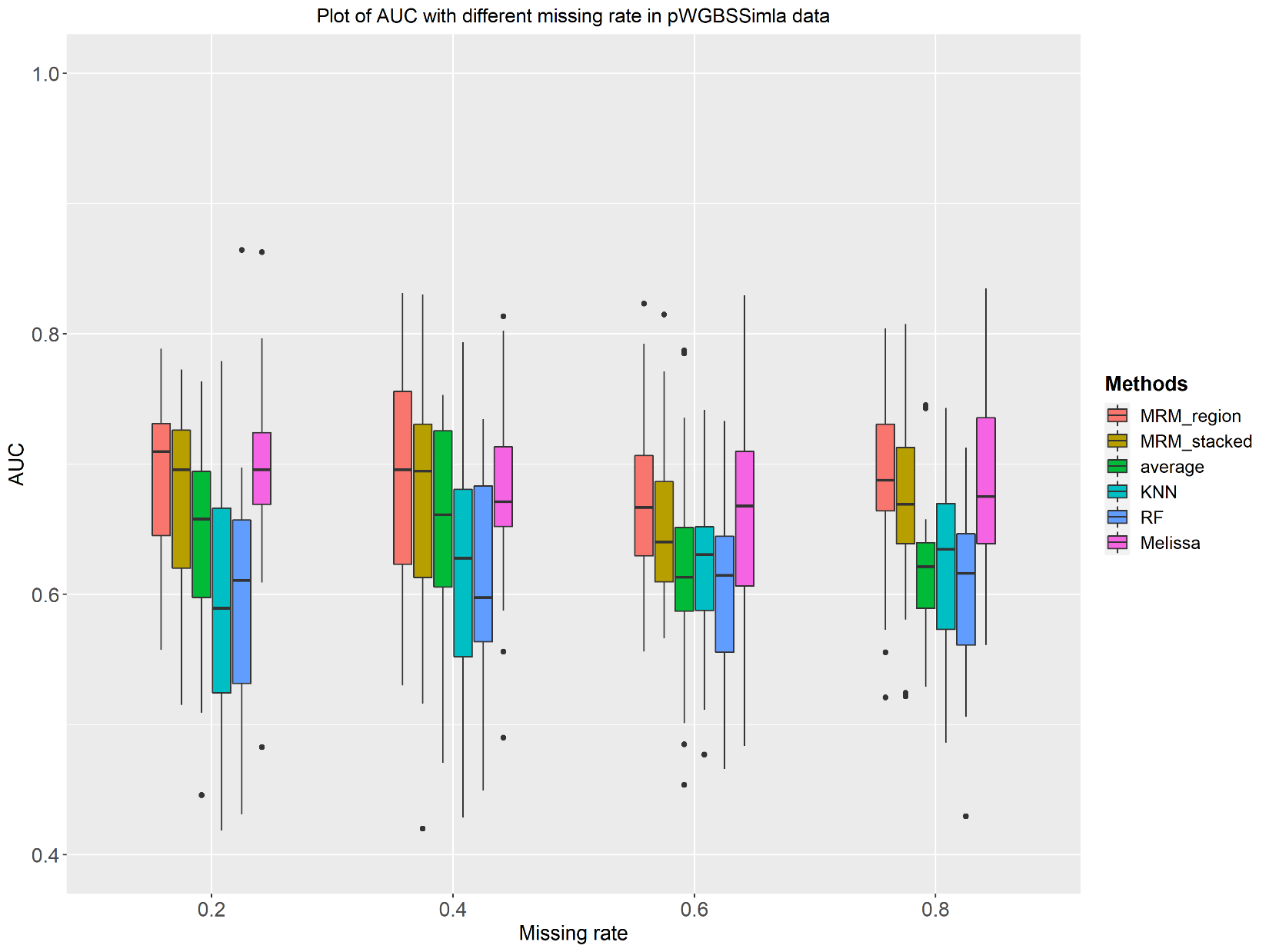


## Figure S1c. Box plot of AUC of six imputation algorithms on pWGBSSimla simulated data under the effect of the missing rate. Each colored boxplot indicates the imputation performance on N=20 subjects.


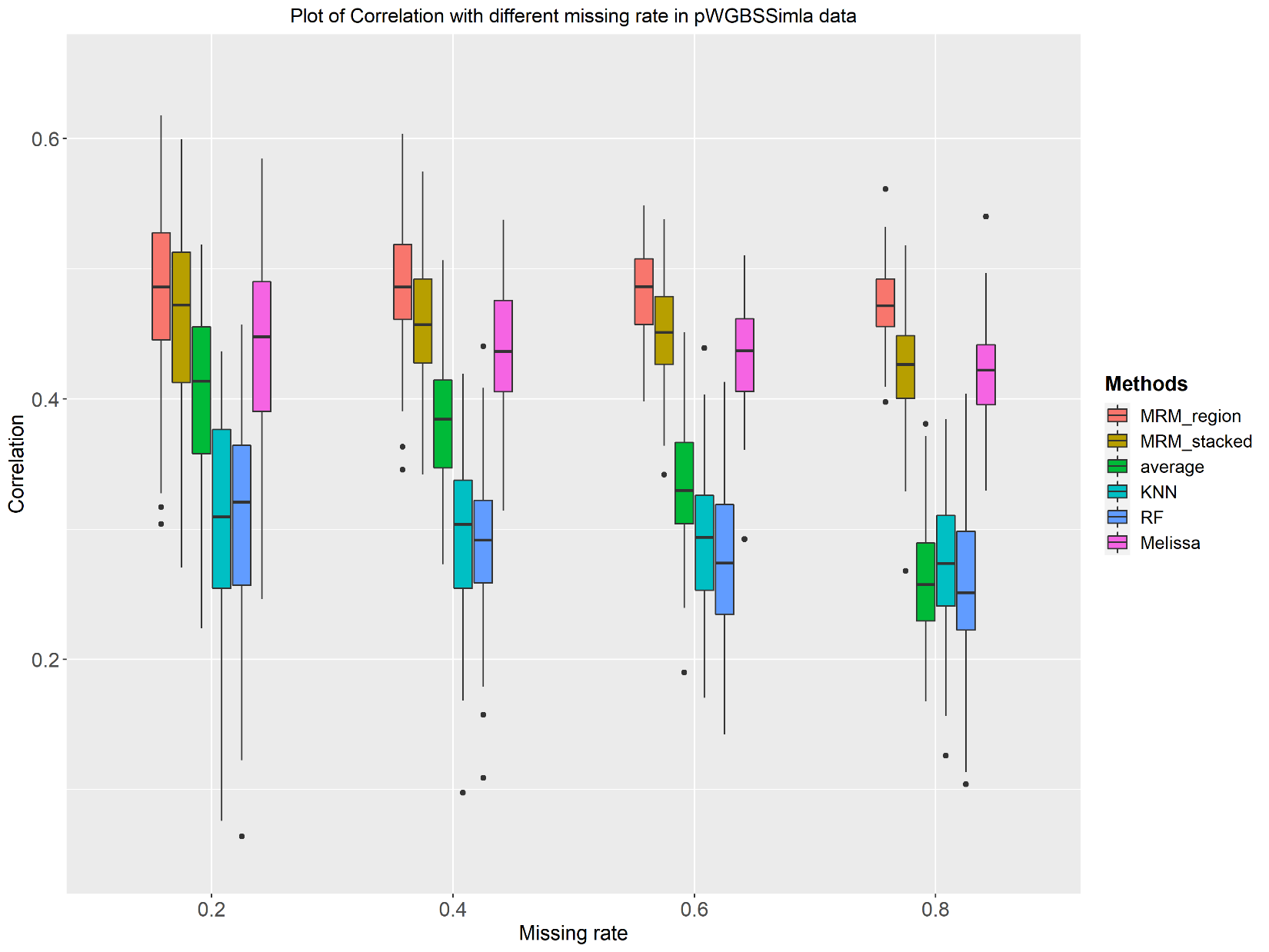


## Figure S1d. Box plot of correlation of six imputation algorithms on pWGBSSimla simulated data under the effect of the missing rate. Each colored boxplot indicates the imputation performance on N=100 subjects.


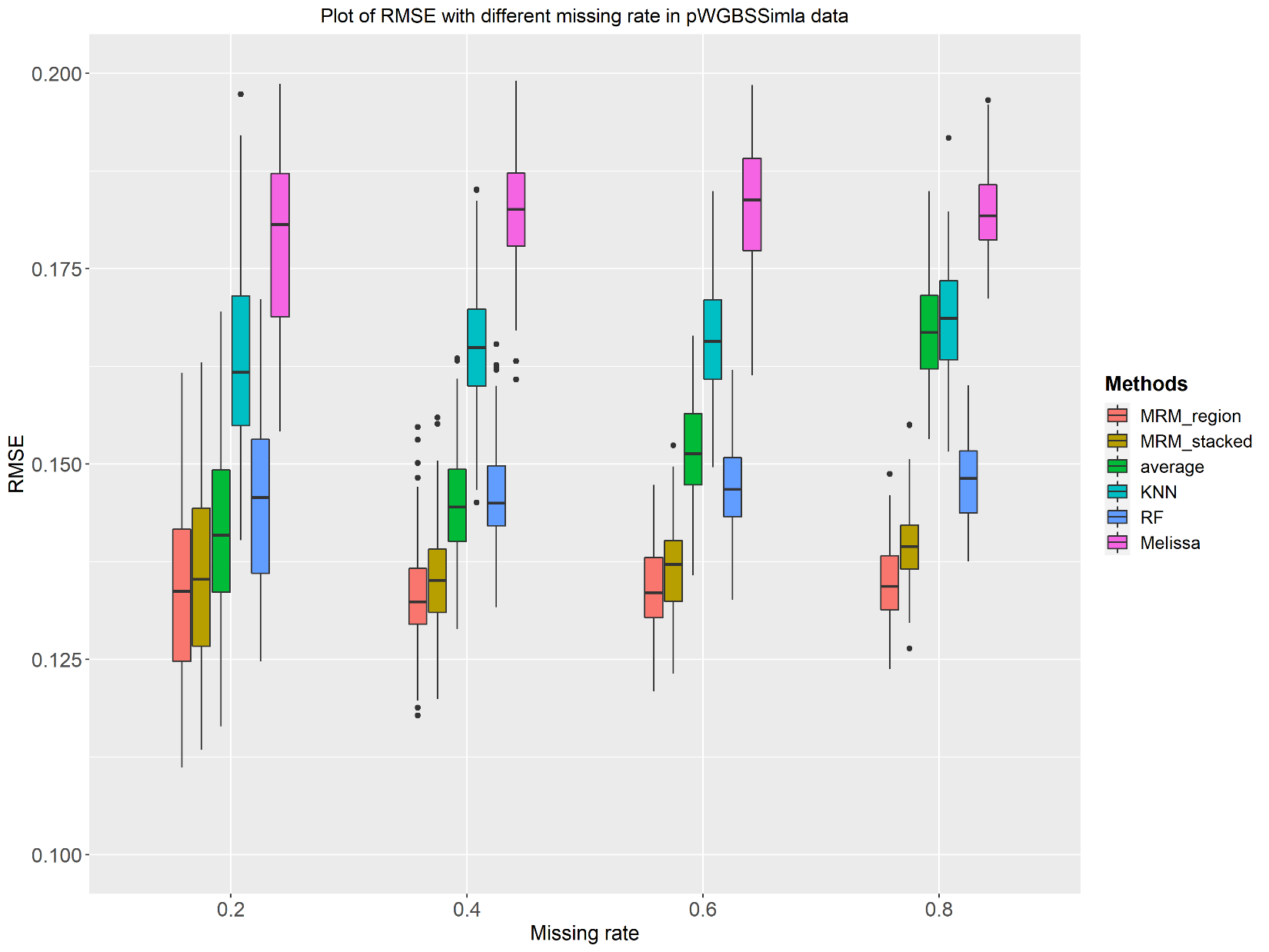


## Figure S1e. Box plot of correlation of six imputation algorithms on pWGBSSimla simulated data under the effect of the missing rate. Each colored boxplot indicates the imputation performance on N=100 subjects.


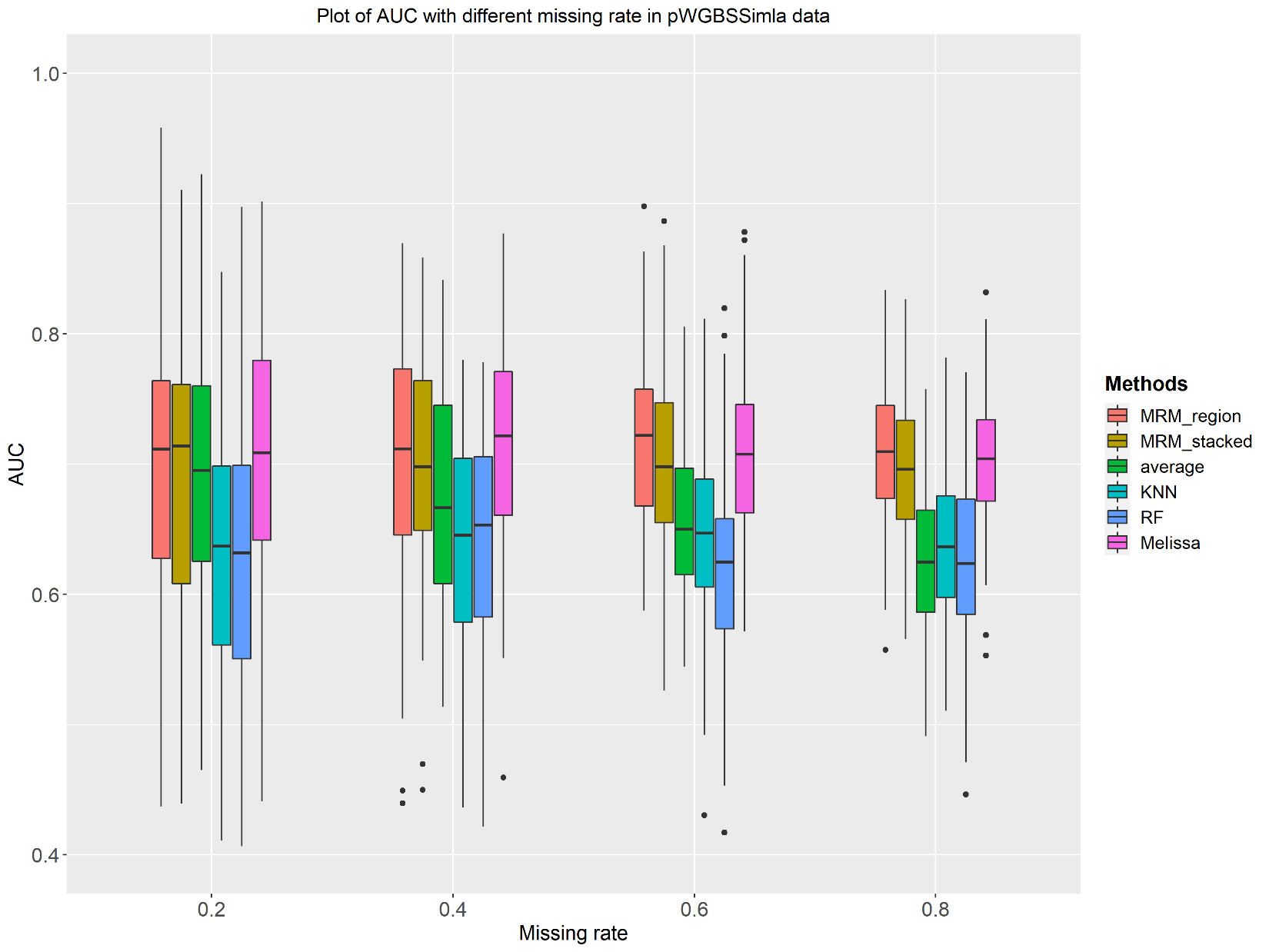


## Figure S1f. Box plot of AUC of six imputation algorithms on pWGBSSimla simulated data under the effect of the missing rate. Each colored boxplot indicates the imputation performance on N=100 subjects.


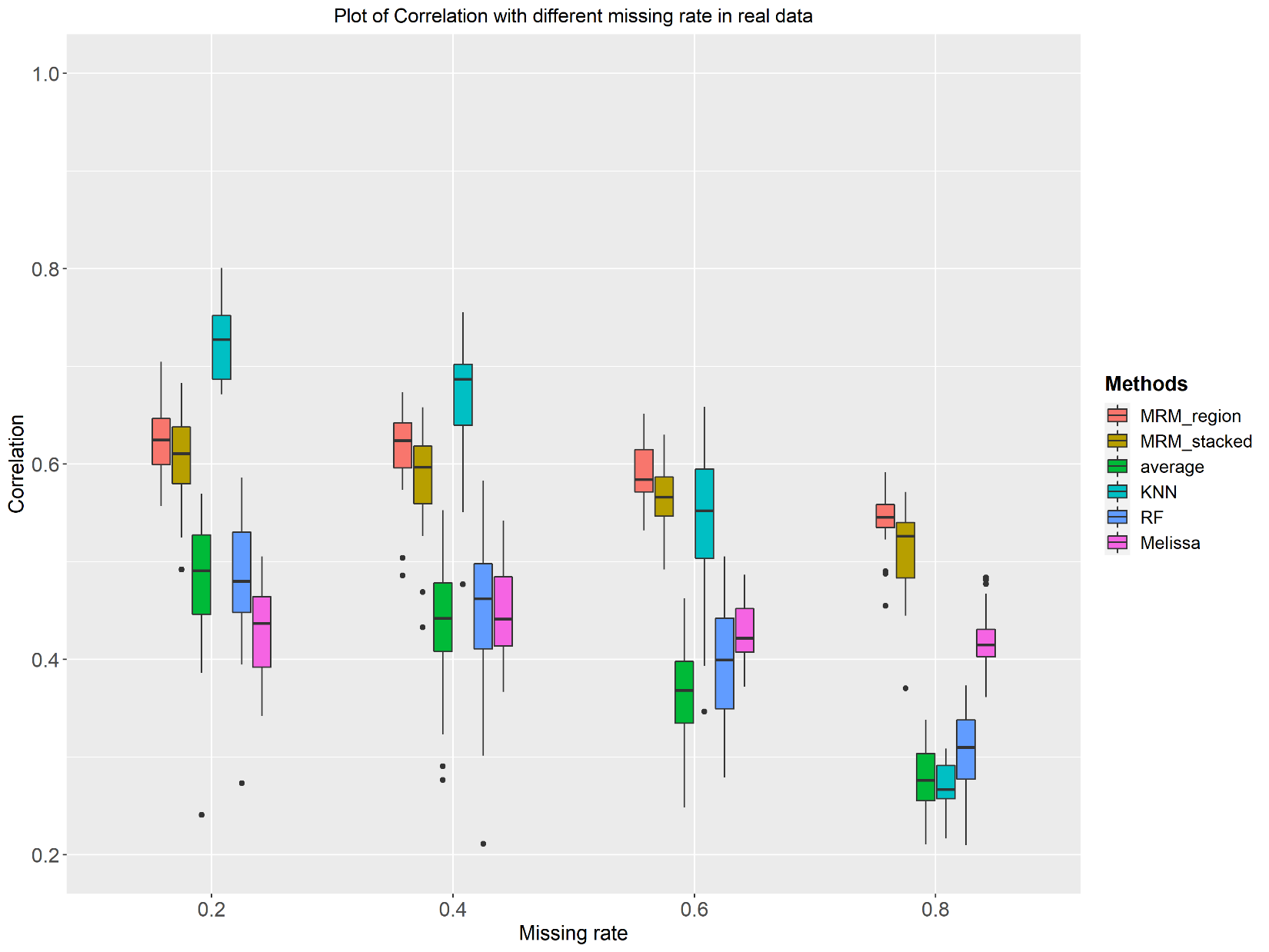


## Figure S2a. Box plot of correlation of six imputation algorithms on real WGBS data under the effect of the missing rate, data preprocess condition 1 (no clustering, no filtering, sliding window). Each colored boxplot indicates the imputation performance on 19 subjects.


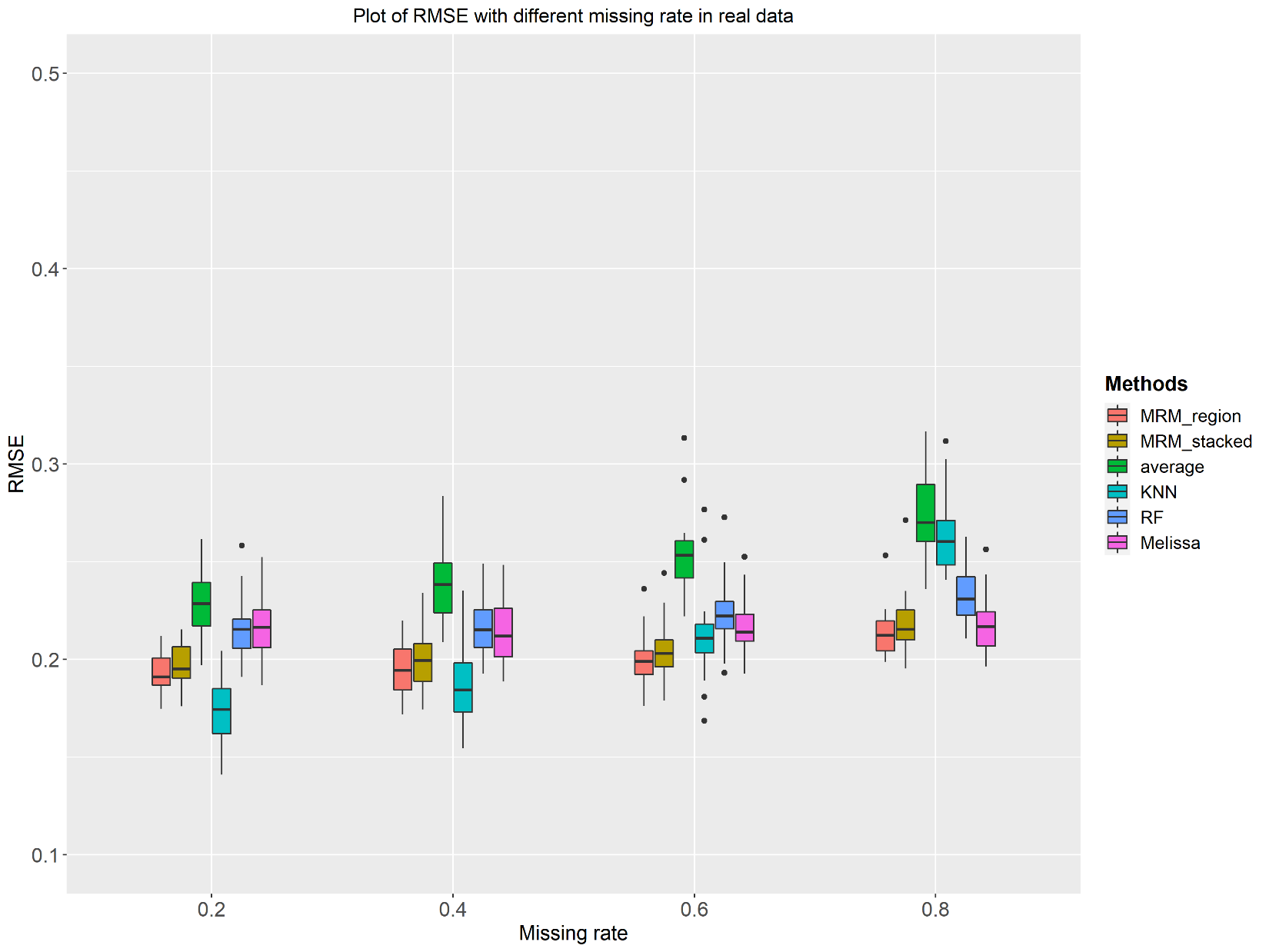


## Figure S2b. Box plot of RMSE of six imputation algorithms on real WGBS data under the effect of the missing rate, data preprocess condition 1 (no clustering, no filtering, sliding window). Each colored boxplot indicates the imputation performance on 19 subjects.


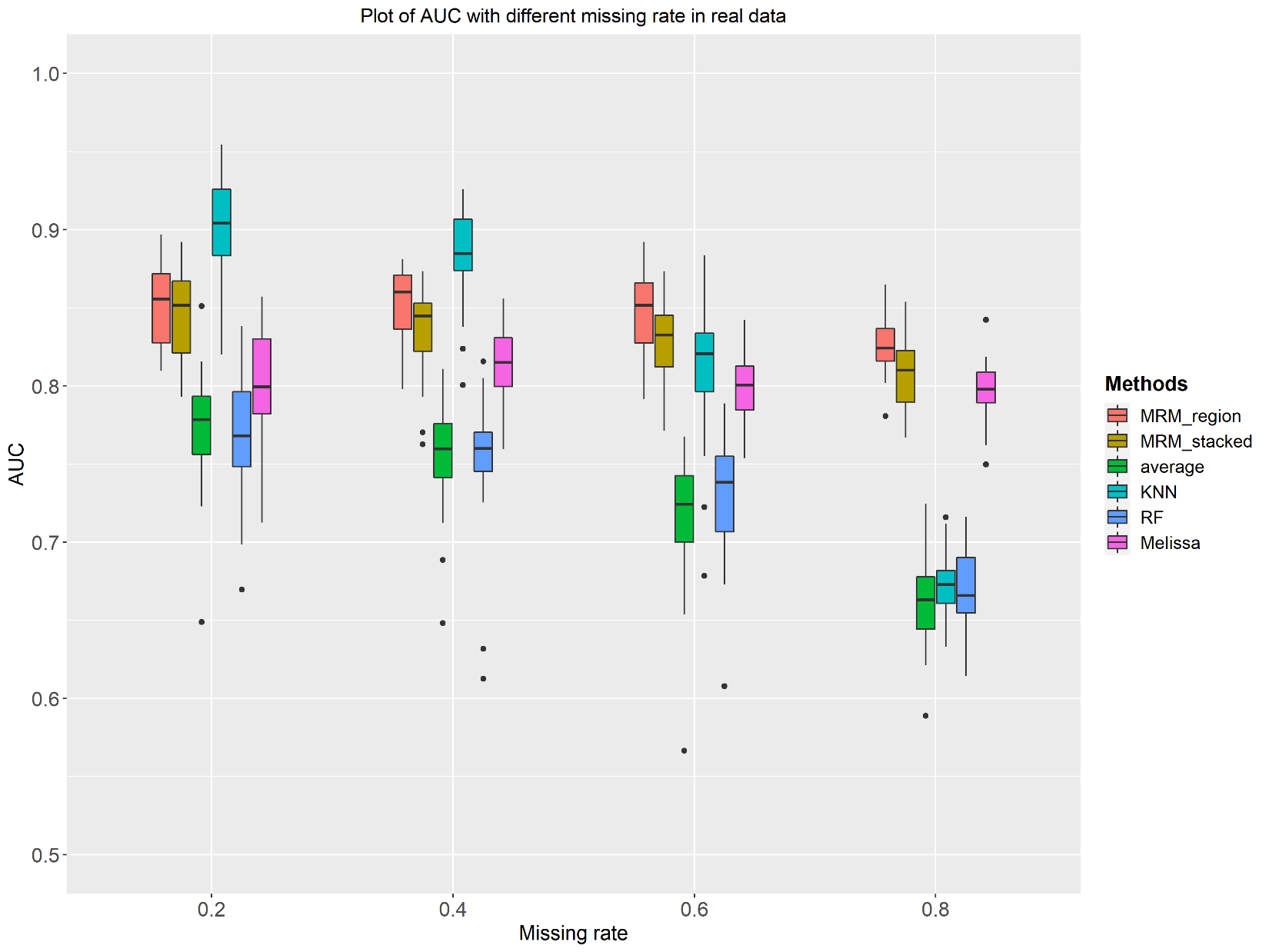


## Figure S2c. Box plot of AUC of six imputation algorithms on real WGBS data under the effect of the missing rate, data preprocess condition 1 (no clustering, no filtering, sliding window). Each colored boxplot indicates the imputation performance on 19 subjects.


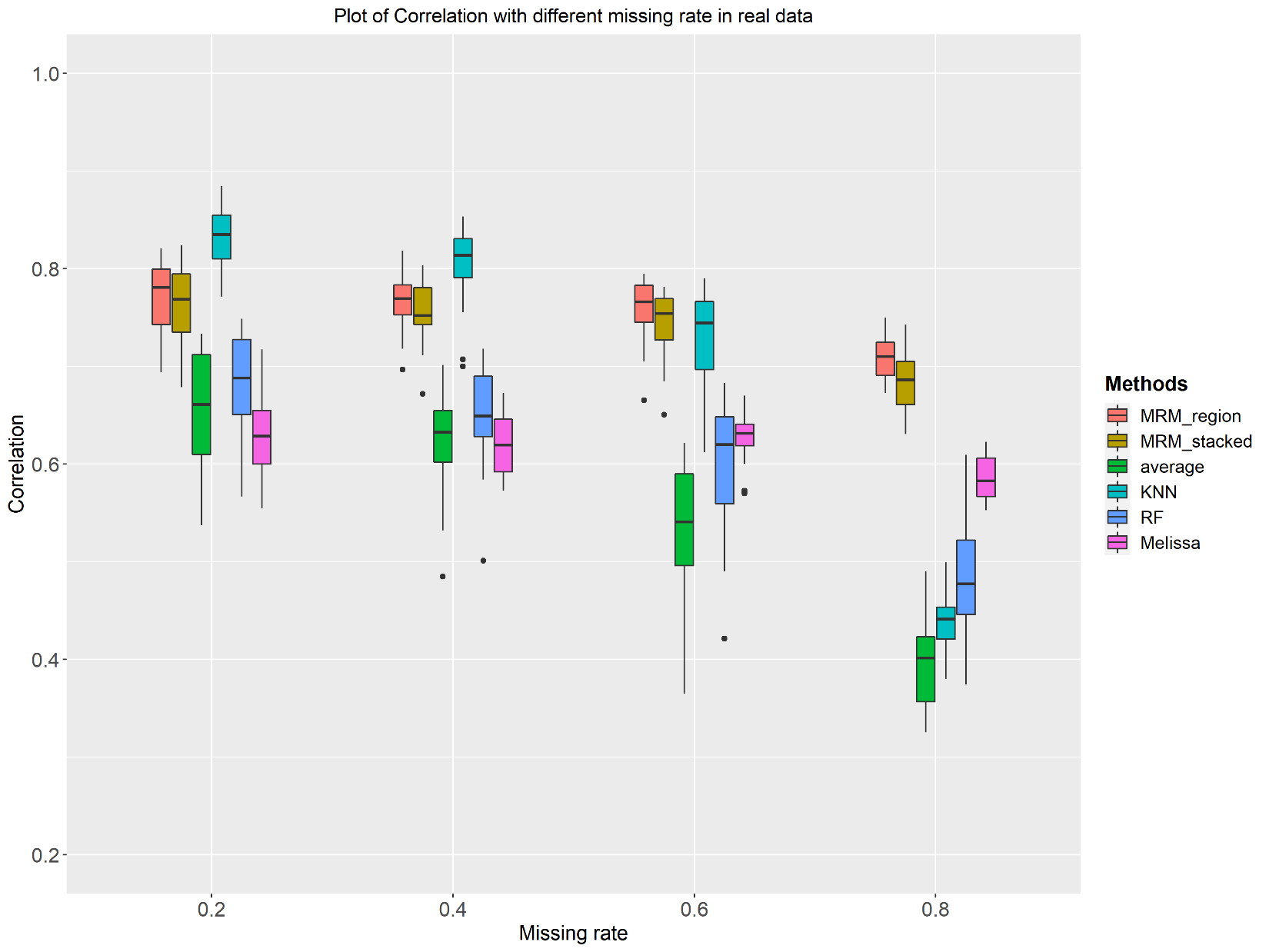


## Figure S3a. Box plot of correlation of six imputation algorithms on real WGBS data under the effect of the missing rate, data preprocess condition 2 (no clustering, no filtering, promoter region). Each colored boxplot indicates the imputation performance on 19 subjects.


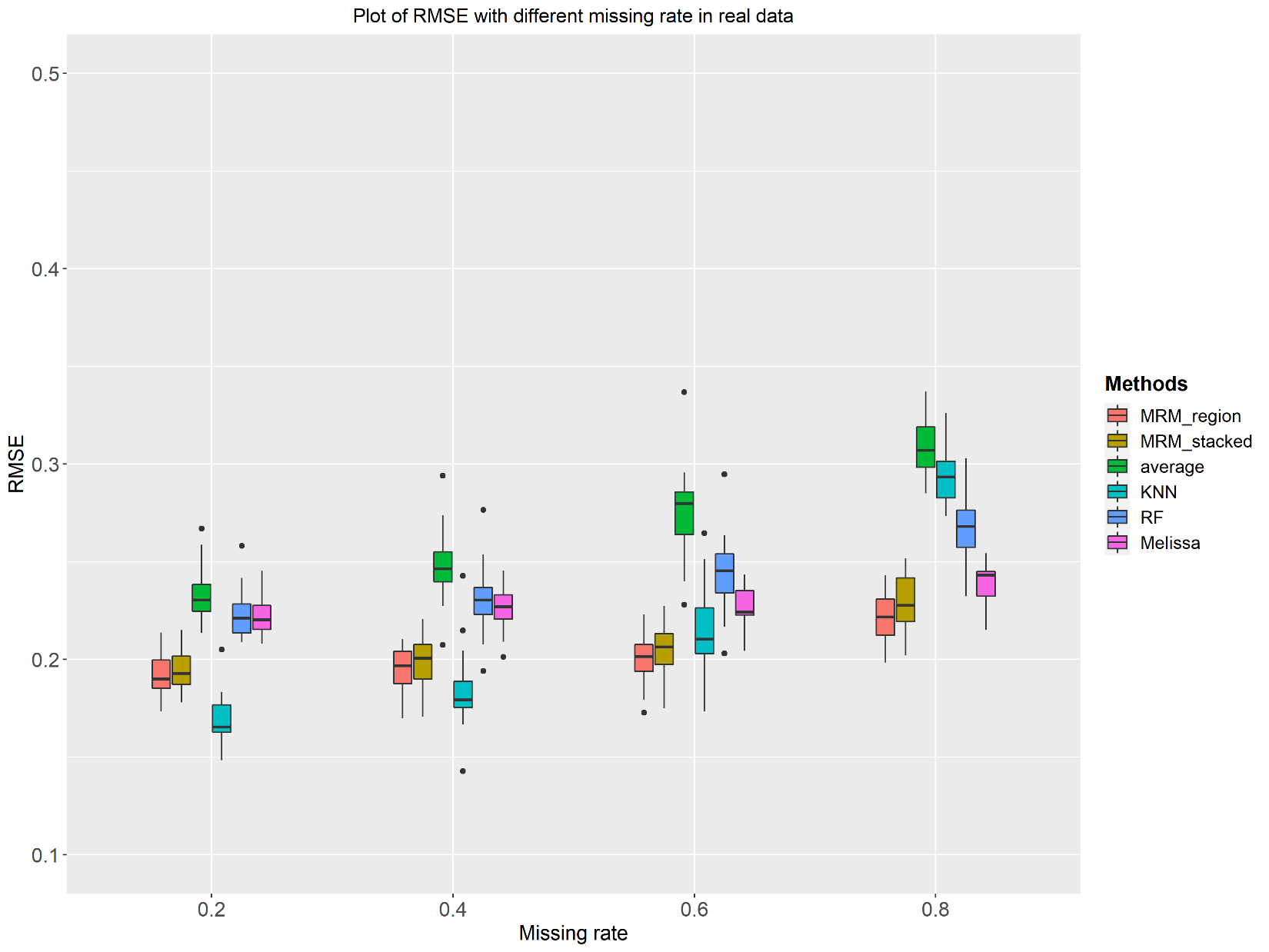


## Figure S3b. Box plot of RMSE of six imputation algorithms on real WGBS data under the effect of the missing rate, data preprocess condition 2 (no clustering, no filtering, promoter region). Each colored boxplot indicates the imputation performance on 19 subjects.


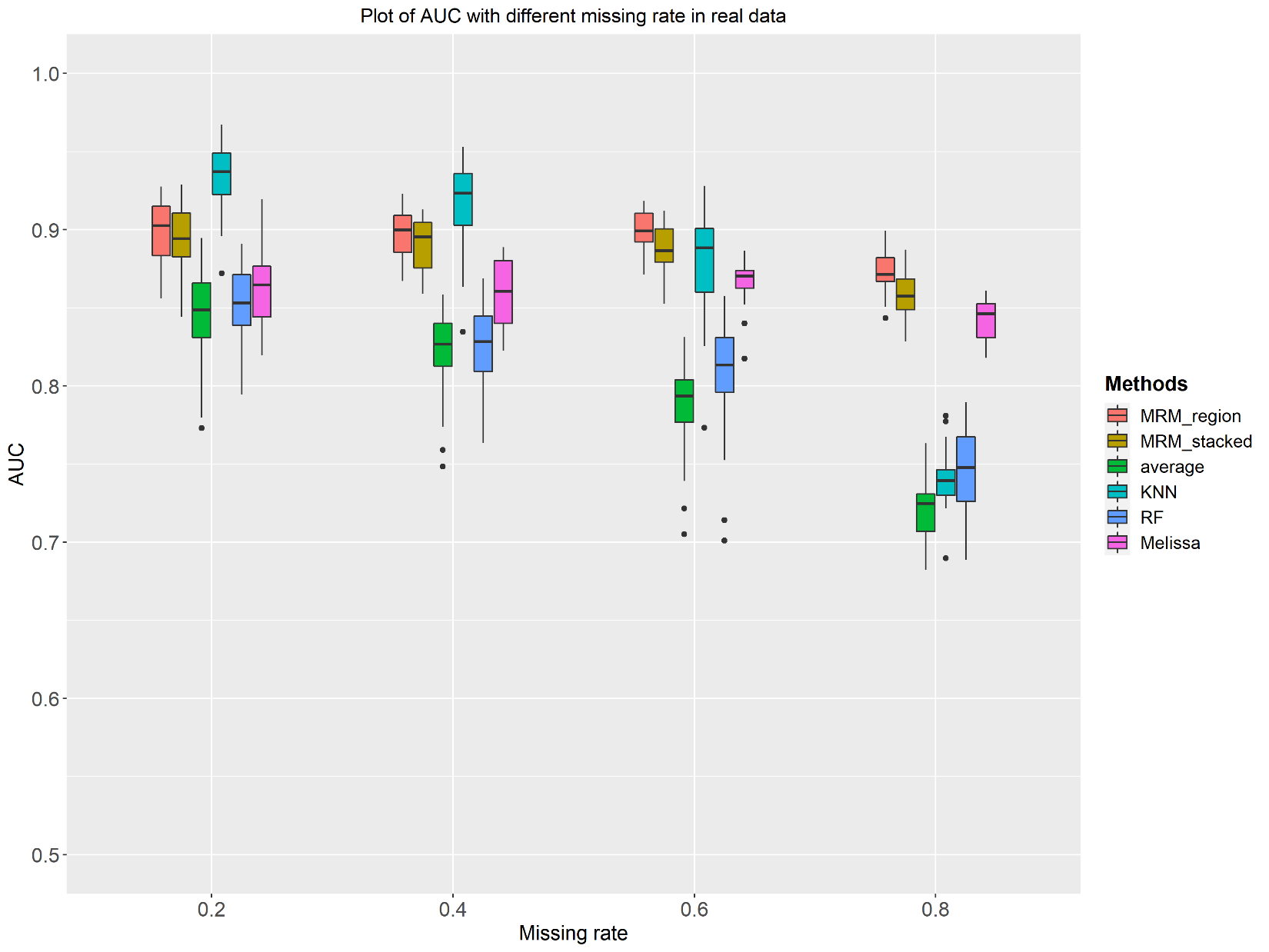


## Figure S3c. Box plot of AUC of six imputation algorithms on real WGBS data under the effect of the missing rate, data preprocess condition 2 (no clustering, no filtering, promoter region). Each colored boxplot indicates the imputation performance on 19 subjects.


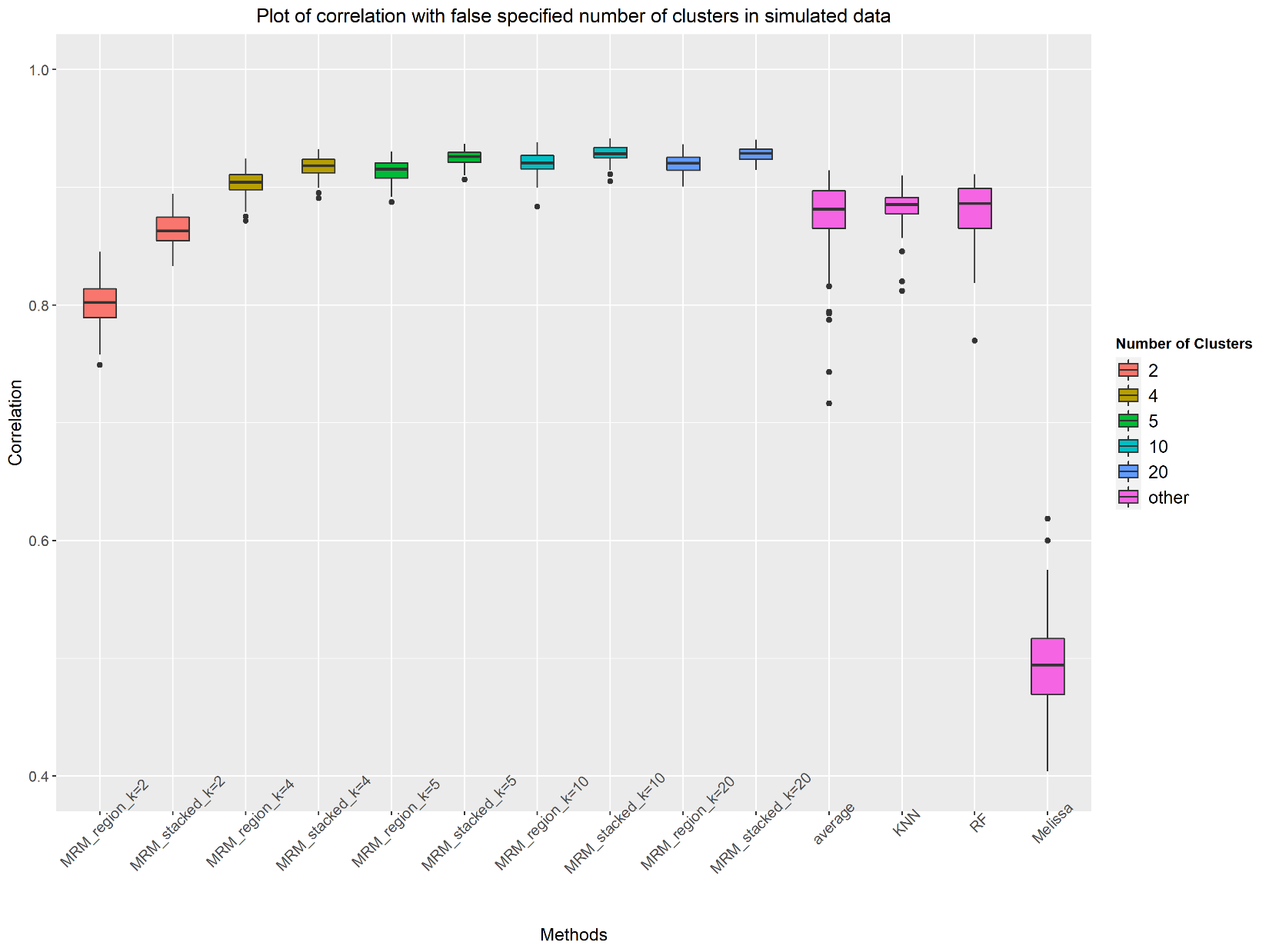


## Figure S4a. Box plot of correlation of MRM with misspecified number of clusters and competing methods in simulated data (true number of clusters = 4, missing rate = 0.2, sample size = 100, variance of Gaussian noise = 0.2).


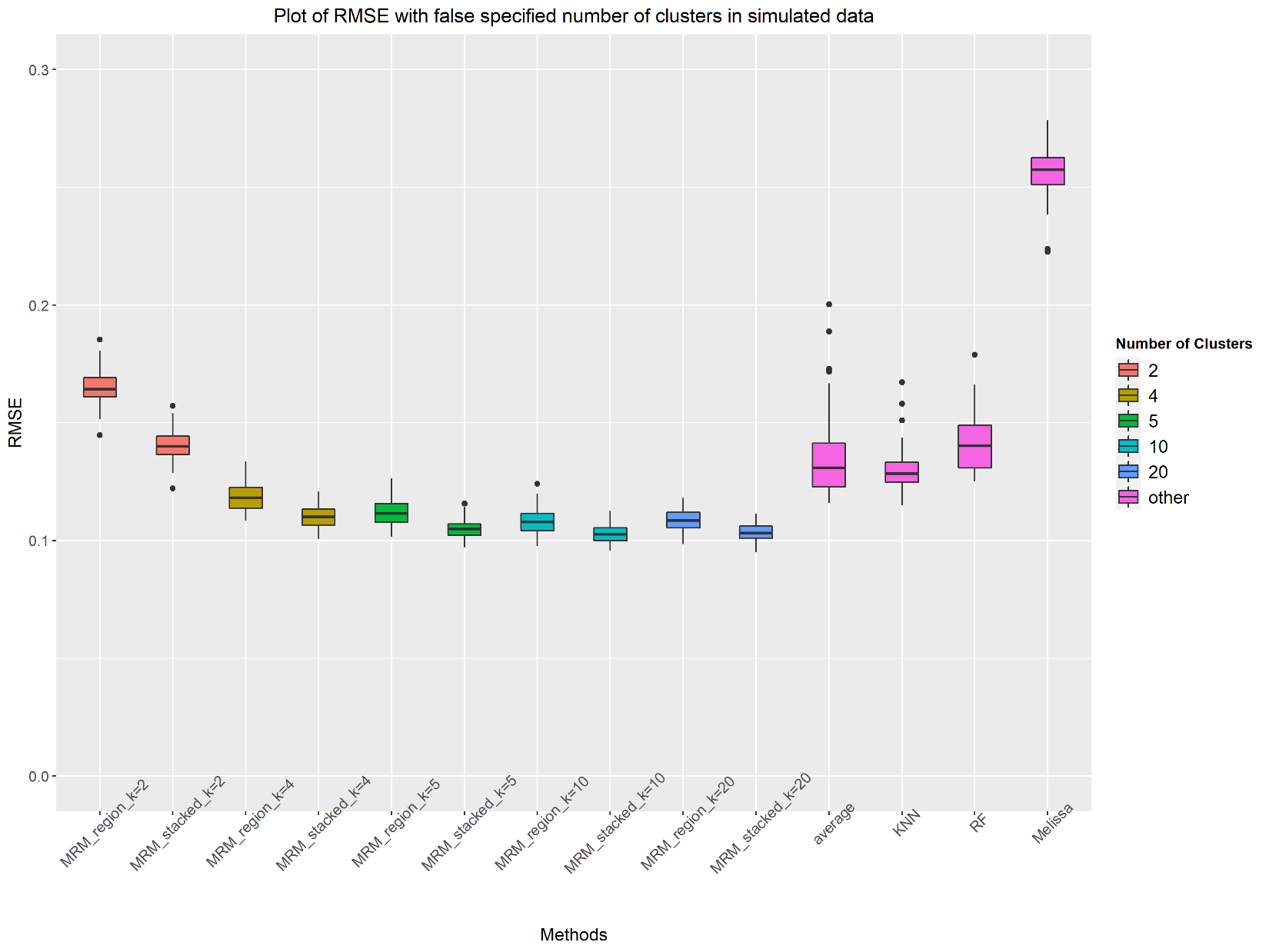


## Figure S4b. Box plot of RMSE of MRM with misspecified number of clusters and competing methods in simulated data (true number of clusters = 4, missing rate = 0.2, sample size = 100, variance of Gaussian noise = 0.2).


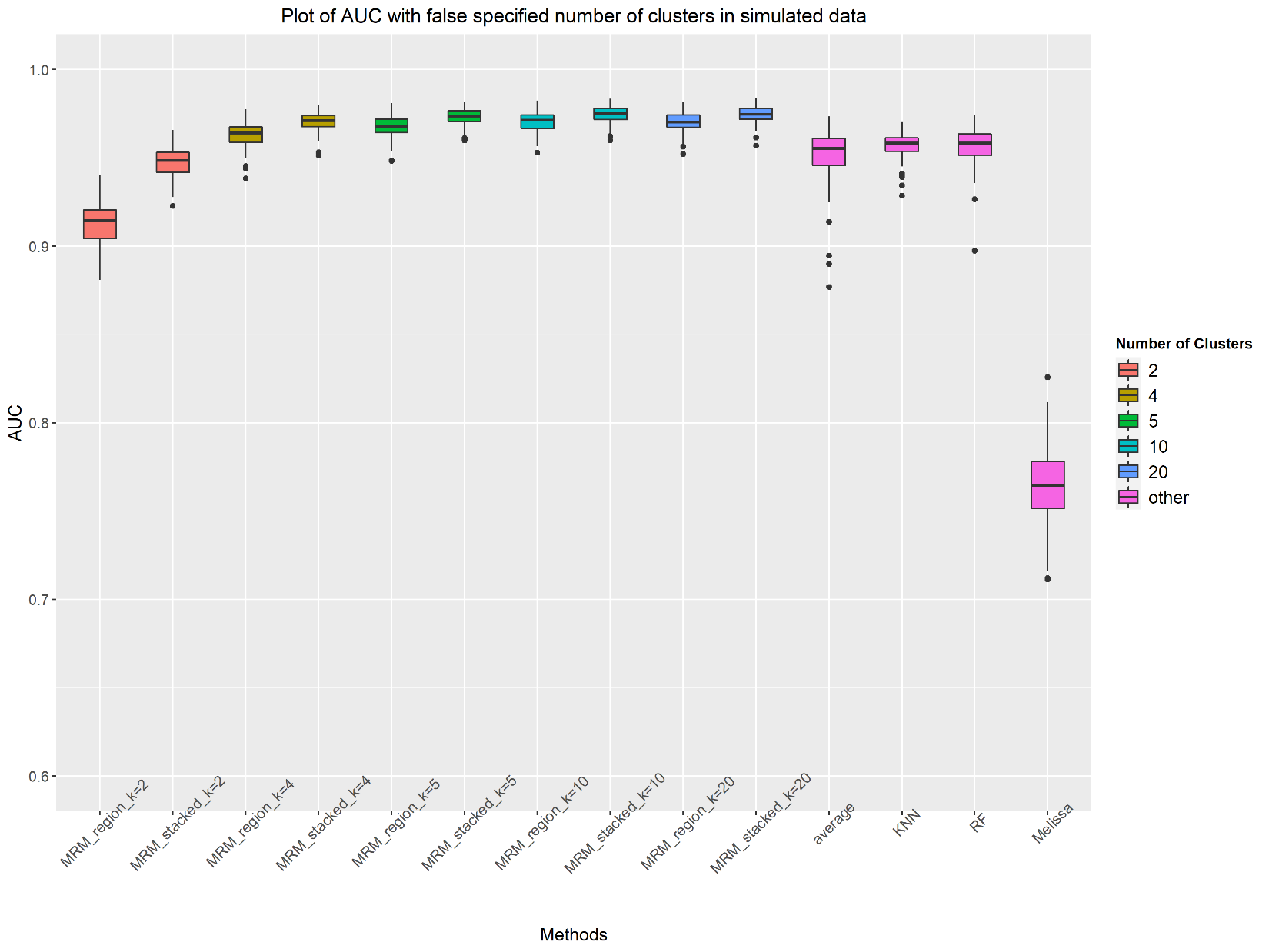


## Figure S4c. Box plot of AUC of MRM with misspecified number of clusters and competing methods in simulated data (true number of clusters = 4, missing rate = 0.2, sample size = 100, variance of Gaussian noise = 0.2).
